# Individual-level Factors Associated With Acute Hospitalization After Medically Attended Acute Gastroenteritis and Norovirus Gastroenteritis in the United States, 2022–2024

**DOI:** 10.1093/ofid/ofag449

**Published:** 2026-07-27

**Authors:** John Shen, Christine Kim, Christopher Bush, Wen-Hsing Wu, Caroline Bridges, Allison E Olmsted, Julia M Baker, Brandon J Patterson, Ben A Lopman, Daniel C Payne, Evan J Anderson, Katherine B Carlson

**Affiliations:** Life Sciences, Datavant, Inc., Phoenix, Arizona, USA; Moderna, Inc., Cambridge, Massachusetts, USA; Life Sciences, Datavant, Inc., Phoenix, Arizona, USA; Moderna, Inc., Cambridge, Massachusetts, USA; Life Sciences, Datavant, Inc., Phoenix, Arizona, USA; Epidemiologic Research and Methods, LLC, Atlanta, Georgia, USA; Epidemiologic Research and Methods, LLC, Atlanta, Georgia, USA; Moderna, Inc., Cambridge, Massachusetts, USA; Epidemiologic Research and Methods, LLC, Atlanta, Georgia, USA; Rollins School of Public Health, Emory University, Atlanta, Georgia, USA; Epidemiologic Research and Methods, LLC, Atlanta, Georgia, USA; Division of Infectious Diseases, Cincinnati Children’s Hospital Medical Center, Cincinnati, Ohio, USA; Moderna, Inc., Cambridge, Massachusetts, USA; Moderna, Inc., Cambridge, Massachusetts, USA

**Keywords:** acute gastroenteritis, acute hospitalization, comorbidity, norovirus, underlying medical condition

## Abstract

**Background:**

Norovirus is a leading cause of acute gastroenteritis (AGE) in the United States and is associated with substantial healthcare utilization, including risk of hospitalization. The extent to which underlying medical conditions contribute to this risk is not well understood.

**Methods:**

This retrospective cohort study analyzed adults with incident medically attended all-cause AGE or cause-specified norovirus AGE episodes identified via *International Classification of Diseases, Tenth Revision, Clinical Modification* between July 1, 2022 and June 30, 2024 in Optum's deidentified Clinformatics® Data Mart Database (Optum® CDM). Generalized estimating equations estimated adjusted risk ratios (aRRs) and confidence intervals (CIs) for acute hospitalization within 3 days of AGE diagnoses.

**Results:**

Of 1 705 514 all-cause AGE and 5805 norovirus AGE cases, 11.3% and 44.6%, respectively, were hospitalized within 3 days of their episode. Hospitalization risk was higher for patients with cardiovascular disease (CVD) (all-cause AGE aRR: 1.67 [95% CI, 1.64–1.69]; norovirus AGE aRR: 1.37 [1.22–1.54]), blood disorders, chronic respiratory disease, and chronic kidney disease. Relative to individuals without underlying conditions, hospitalization risk was higher for those with ≥2 conditions (all-cause AGE aRR: 2.02 [95% CI, 1.98–2.07]; norovirus AGE aRR: 1.79 [1.50–2.14]), exceeding the risk among those with 1 condition. Relative risk of acute hospitalization associated with presence of underlying conditions was higher among adults aged 18–64 years than among those ≥65 years.

**Conclusions:**

Underlying conditions, notably CVD and ≥2 underlying conditions, significantly increased the risk of acute hospitalization, which was elevated among adults aged 18–64 years.

## Teaser

The presence of underlying medical conditions, particularly cardiovascular disease and ≥2 underlying conditions, increased hospitalization risk after acute gastroenteritis. Older adults had the highest absolute hospitalization risk, but younger adults with underlying conditions experienced a greater relative increase in hospitalization.

Norovirus is the leading cause of acute gastroenteritis (AGE) in the United States (US), responsible for ∼2.3 million outpatient visits, 470 000 emergency department (ED) visits, and 110 000 hospitalizations annually [1]. While most norovirus infections are self-limiting, older adults and those with underlying medical conditions are particularly susceptible to complications such as severe dehydration, malnutrition, and acute renal failure [2, 3]. Adults ≥65 years of age account for an estimated 40% of norovirus-related hospital admissions and 90% of norovirus-associated deaths in the US [4, 5].

One US Veterans Health Administration study demonstrated that patients with laboratory-confirmed norovirus infection had a 13% higher risk of acute hospitalization within 3 days and increased risk of short-term mortality compared with those with norovirus-negative AGE [3]. In an analysis of Merative MarketScan claims data, individuals with underlying conditions such as cardiovascular disease (CVD), chronic kidney disease (CKD), and immunocompromising conditions had increased annual rates of all-cause AGE and norovirus AGE-associated hospitalizations [6]. However, this analysis was limited to patients with a single condition, which may not adequately describe the estimated 51.4% of US adults reporting multiple comorbidities [7]. Additionally, this study examined only cause-specified hospitalizations. Given known limitations in testing and documentation, along with the lack of robust reimbursement for AGE-related treatment [8], these codes likely underestimate the true burden of hospitalization in these high-risk groups.

Other studies exploring the role of underlying conditions in norovirus AGE hospitalizations and clinical outcomes are limited by small sample sizes or a lack of contemporaneous data [9–11]. The risk of acute hospitalization and the role of underlying conditions among individuals in the US with norovirus AGE remains understudied, particularly in the post-COVID-19 pandemic period. The objective of this noninterventional, real-world study was to describe the frequency of acute hospitalization within 3 days of all-cause AGE or cause-specified norovirus AGE diagnosis and determine associations between patient-level characteristics, including age and presence of underlying medical conditions.

## METHODS

### Study Design and Population

We conducted a retrospective cohort study using Optum's deidentified Clinformatics® Data Mart Database (Optum® CDM). The study population included adults aged ≥18 years with diagnosis of AGE between July 1, 2022 and June 30, 2024 (ie, the cohort entry period), identified via *International Classification of Diseases, Tenth Revision, Clinical Modification* (ICD-10-CM) codes on a medical claim. Analyses were conducted separately within 2 cohorts that were not mutually exclusive: (1) individuals with diagnoses representing all-cause AGE (Supplementary Table 1); (2) a subset of individuals with cause-specified norovirus AGE (ICD-10-CM: A08.1*). For each cohort, claims with AGE diagnosis codes within 14 days of one another were grouped into a single episode, with the index date set to the first claim. A patient could enter the cohort multiple times if they had more than 1 episode during the cohort entry period that met all eligibility criteria, including at least 12 months of continuous enrollment before each index date, with 30-day gaps allowed (Supplementary Figure 1).

### Data Source

Optum's deidentified Clinformatics® Data Mart (Optum® CDM or Optum Clinformatics®) is derived from a database of administrative health claims for members of large commercial and Medicare Advantage health plans. Optum Clinformatics® utilizes medical, and pharmacy claims to derive patient-level enrollment information, health care costs, and resource utilization information. The population is geographically diverse, spanning all 50 states and is statistically deidentified under the Health Insurance Portability and Accountability Act Privacy Rule's Expert Determination method and managed according to Optum® customer data use agreements. Optum® CDM administrative claims submitted for payment by providers and pharmacies are verified, adjudicated and deidentified prior to inclusion.

### Patient Consent Statement

As this study relied on deidentified secondary data without direct patient contact, institutional review board approval, and informed consent were not required.

### Analysis

Demographic characteristics were assessed on the index date. The following study-defined underlying medical conditions were evaluated as ≥1 inpatient claim or ≥2 outpatient claims in the 365-day baseline period prior to index date: blood disorders, CVD, chronic respiratory disease, chronic liver disease, CKD, diabetes, obesity, neurological disorders, chronic gastrointestinal disease, and chronic immunocompromising disorders. These conditions were identified via investigator review of relevant diagnoses, procedure codes, and medications (Supplementary Table 1). The Charlson-Quan Comorbidity Index (CCI) was also included as a standardized measure of comorbidity [12]. The clinical setting of all-cause AGE or norovirus AGE diagnosis was reported on the index date. If healthcare encounters occurred in multiple clinical settings on the same day, the setting was categorized based on the highest level of care received, prioritized in the following order: inpatient, ED, outpatient. Categorical variables were summarized using counts and percentages (*n*, %). Continuous variables were summarized using means, standard deviations (SD), medians, and interquartile range (IQR). Because patients could contribute multiple AGE episodes, the episode served as the primary unit of observation for all analyses.

Across study cohorts, acute hospitalization was characterized by frequency, length of stay (LOS), and proportion requiring intensive care unit (ICU) admission during an acute hospitalization stay. Acute hospitalization was defined as any claim in the inpatient setting within 3 days of follow-up on or after the index date and assessed as all-cause hospitalization. Other healthcare resource utilization (HCRU) events, including the use of antiemetics, antidiarrheal agents, and antibiotics, were identified via procedure codes and relevant medications within 3 days on or after the index date (Supplementary Table 1).

Generalized estimating equations (GEE) with a log link and an independent correlation structure, clustered by patient to account for repeated measures, were used to estimate adjusted risk ratios (aRRs) and 95% confidence intervals (CIs) for acute hospitalization separately among patients with all-cause AGE or norovirus AGE, after adjusting for age group, sex, and region. Separate GEE models were used to assess the association between the outcome of acute hospitalization and the presence of any underlying condition, the presence of specific underlying conditions (adjusting for other conditions), number of underlying conditions (0, 1, ≥2 conditions), and CCI score (0, 1, ≥2). Multimorbidity was defined as the presence of ≥2 study-defined conditions or a baseline CCI score of ≥2. Associations within age-stratified groups, 18–64 years and ≥65 years, were also reported, adjusted for sex and region.

Analyses were performed using Aetion Substantiate version 5.62.0 and R version 4.5.1.

## RESULTS

### Study Population

A total of 1 705 514 all-cause AGE episodes and 5805 cause-specified norovirus AGE episodes were identified between July 1, 2022 and June 30, 2024 (Table 1). On average, adult patients in the all-cause AGE and norovirus AGE cohorts contributed 1.53 and 1.08 episodes, respectively. The proportion of all-cause AGE and norovirus AGE episodes with an encounter in the inpatient setting on index date was 9.7% and 42.8%, respectively. Demographics were broadly consistent across both cohorts (Table 1). Patients in the all-cause AGE cohort had a median (IQR) age of 69 (54–77) years, while those in the norovirus AGE cohort had a median age of 72 (58–80) years. In both cohorts, most patients were female (65.1% and 59.0%, respectively), White (66.6% and 70.3%), and from the South (44.8% and 39.8%). Most individuals had at least one underlying condition in the baseline period, 82.4% of the all-cause AGE cohort and 87.7% of the norovirus AGE cohort. Patients with all-cause AGE had a mean (SD) of 2.6 (2.0) study-defined underlying conditions with a CCI score of 2.0 (2.4), and those with norovirus AGE had 3.3 (2.2) underlying conditions and a CCI score of 2.8 (2.7).

**Table 1.** Demographics and Baseline Clinical Characteristics of All-cause AGE and Norovirus AGE Episodes

	All-cause AGE	Norovirus AGE
Total number of eligible episodes^a^	1 705 514	5805
Unique patients		
Total number of unique adult patients	1 112 202	5367
Episodes per unique adult patient	1.53	1.08
Number of unique adult patients with >1 eligible episodes; n (%)	313 519 (28.2%)	293 (7.3%)
Episode characteristics		
Episode setting; n (%)		
Outpatient	1 278 613 (75.0%)	2130 (36.7%)
ED	260 914 (15.3%)	1193 (20.6%)
Inpatient	165 987 (9.7%)	2482 (42.8%)
Number of separate encounters within the episode; n (%)		
1	1 392 702 (81.7%)	4846 (83.5%)
≥2	312 812 (18.3%)	959 (16.5%)
Duration of episode, days		
Mean (SD)	4.4 (11.7)	7.1 (13.9)
Median [IQR]	1 [1–3]	3 [1–7]
Demographics		
Age, years		
Mean (SD)	64.2 (17.8)	66.9 (17.8)
Median [IQR]	69 [54–77]	72 [58–80]
Age group, years; n (%)		
18–59	527 013 (30.9%)	1535 (26.4%)
60–64	114 619 (6.7%)	376 (6.5%)
65–69	228 509 (13.4%)	684 (11.8%)
70–74	266 894 (15.6%)	789 (13.6%)
75–79	244 200 (14.3%)	812 (14.0%)
≥80	324 279 (19.0%)	1609 (27.7%)
Sex; n (%)		
Female	1 110 605 (65.1%)	3427 (59.0%)
Male	593 895 (34.8%)	2376 (40.9%)
Missing/unknown	1014 (0.1%)	2 (<0.1%)
Race/ethnicity; n (%)		
White	1 136 271 (66.6%)	4079 (70.3%)
Black	150 619 (8.8%)	456 (7.9%)
Asian	38 174 (2.2%)	139 (2.4%)
Missing/unknown	380 450 (22.3%)	1131 (19.5%)
Region^b^; n (%)		
Midwest	384 791 (22.6%)	1637 (28.2%)
Northeast	205 692 (12.1%)	843 (14.5%)
South	763 990 (44.8%)	2312 (39.8%)
West	347 589 (20.4%)	1006 (17.3%)
Other	30 (<0.1%)	0 (<0.1%)
Missing/unknown	3422 (0.2%)	7 (0.1%)
Clinical characteristics		
Underlying medical condition risk group; n (%)		
Presence	1 405 050 (82.4%)	5093 (87.7%)
Absence	300 464 (17.6%)	712 (12.3%)
Individual underlying medical conditions; n (%)		
Blood disorders	71 114 (4.2%)	445 (7.7%)
CVD	1 077 510 (63.2%)	4312 (74.3%)
Chronic respiratory disease	528 969 (31.0%)	2358 (40.6%)
Chronic liver disease	137 827 (8.1%)	665 (11.5%)
CKD	320 852 (18.8%)	1785 (30.7%)
Diabetes (types I and II)	471 175 (27.6%)	1867 (32.2%)
Obesity	325 879 (19.1%)	1258 (21.7%)
Neurological disorders	437 544 (25.7%)	2163 (37.3%)
Chronic gastrointestinal disease	682 333 (40.0%)	2530 (43.6%)
Chronic immunocompromising disorders^c^	383 246 (22.5%)	1759 (30.3%)
Total number of underlying medical conditions, continuous		
Mean (SD)	2.6 (2.0)	3.3 (2.2)
Median [IQR]	2 [1–4]	3 [2–5]
Total number of underlying medical conditions, categorical; n (%)		
0	300 464 (17.6%)	712 (12.3%)
1	289 179 (17.0%)	711 (12.2%)
2–3	588 469 (34.5%)	1792 (30.9%)
≥4	527 402 (30.9%)	2590 (44.6%)
Charlson-Quan comorbidity index, continuous		
Mean (SD)	2.0 (2.4)	2.8 (2.7)
Median [IQR]	1 [0–3]	2 [0–4]
Charlson-Quan comorbidity index, categorical; n (%)		
0	663 825 (38.9%)	1532 (26.4%)
1	261 800 (15.4%)	715 (12.3%)
2–3	411 084 (24.1%)	1617 (27.9%)
≥4	368 805 (21.6%)	1941 (33.4%)

Abbreviations: AGE, acute gastroenteritis; ED, emergency department; HIV, human immunodeficiency virus; IQR, interquartile range; SD, standard deviation.

^a^Index date is the first day of an episode of all-cause AGE or norovirus AGE. Demographics are assessed on index date. Clinical characteristics are assessed from 365 d prior to index through 1 d prior to index (ie, baseline period). Unless otherwise specified, all percentages are calculated using the total number of eligible episodes as the denominator.

^b^States are classified into each region according to the US census bureau. The “other” category includes only Puerto Rico.

^c^Chronic immunocompromising disorders were defined using ICD-10-CM codes for acquired hemolytic anemia; aplastic and pure red cell aplasia; central nervous system inflammatory diseases; HIV; inflammatory bowel disease; primary and secondary immunodeficiency disorders; rheumatic autoimmune and inflammatory conditions; solid organ and bone marrow transplantation; and trisomy 21. Certain conditions were further classified in combination with chemotherapy or immunosuppressant use.

### Healthcare Resource Utilization

**Figure 1.**
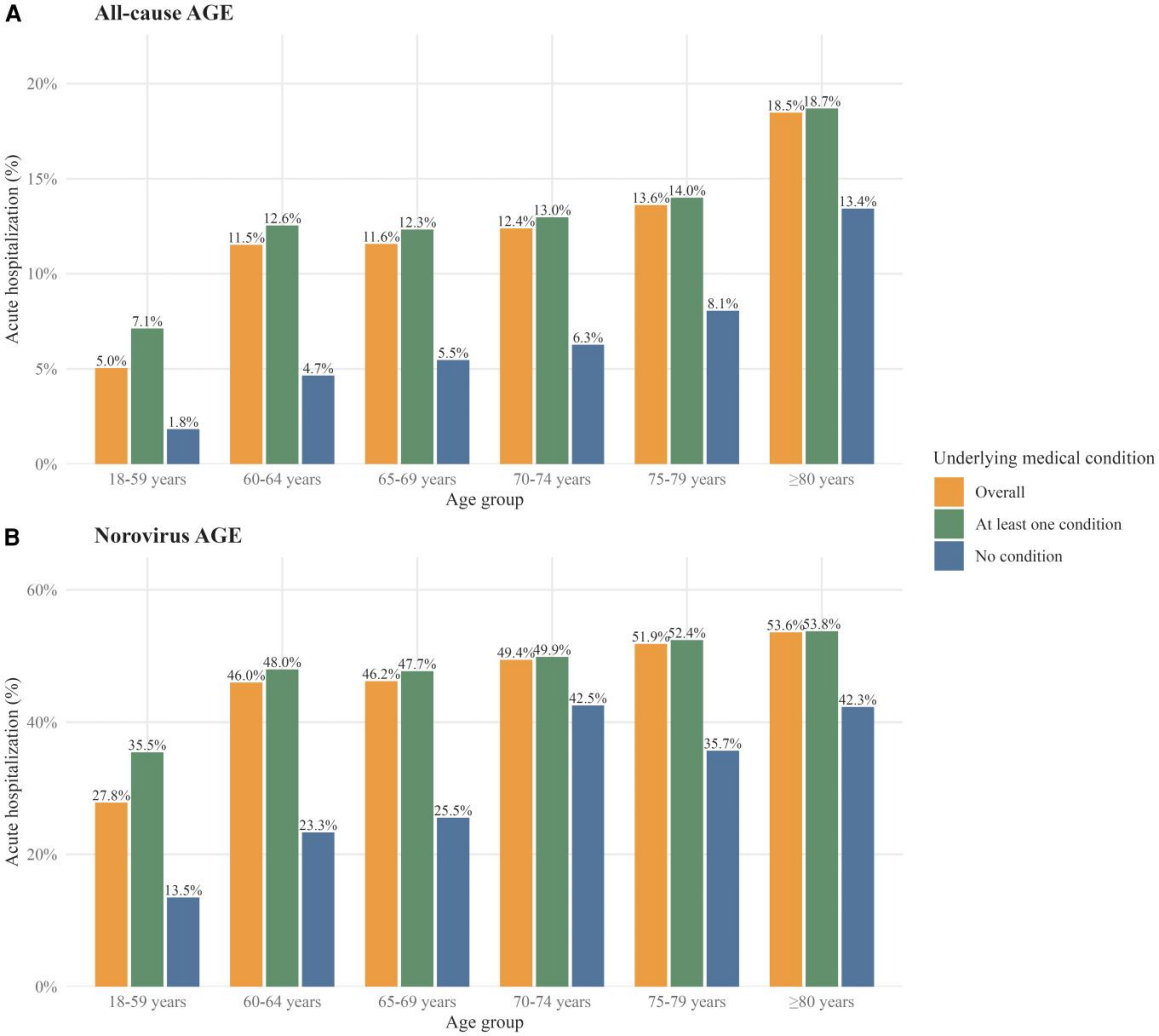
The proportion of all-cause AGE episodes (*A*) and norovirus AGE episodes (*B*) with an acute hospitalization increased with age. Across all age groups, hospitalization was more frequent for episodes with at least one underlying medical condition (green) than for episodes with no underlying condition (blue). Orange bars indicate the total proportion of episodes with acute hospitalization, unstratified by underlying medical condition status. Abbreviation: AGE, acute gastroenteritis.

The proportion of all-cause AGE and norovirus AGE episodes with acute hospitalization within 3 days of index was 11.3% and 44.6%, respectively (Table 2). Among those experiencing acute hospitalization, the proportion requiring ICU admission was 38.4% in the all-cause AGE cohort and 34.2% in the norovirus AGE cohort. For all-cause AGE and norovirus AGE, the frequency of acute hospitalization increased with age, and within each age group, hospitalizations were more frequent for episodes with presence of underlying conditions (Figure 1).

**Table 2.** Acute Hospitalization and Other HCRU Within 3 Days on or After All-cause AGE and Norovirus AGE Episodes

	All-cause AGE				Norovirus AGE			
	Overall	With Underlying Medical Condition	Without Underlying Medical Condition	*P* Value^a^	Overall	With Underlying Medical Condition	Without Underlying Medical Condition	*P* Value^a^
Total number of unique adult patients, n	1 112 202	884 682	259 559	-	5367	4666	708	-
Total number of eligible episodes^b^	1 705 514	1 405 050	300 464	-	5805	5093	712	-
HCRU								
Acute hospitalization within 3 d on or after index date								
Acute hospitalization; n (%)	192 430 (11.3%)	181 967 (13.0%)	10 463 (3.5%)	<.001	2589 (44.6%)	2457 (48.2%)	132 (18.5%)	<.001
LOS^c^	…	…	…	.782	…	…	…	<.001
Mean (SD)	7.3 (9.1)	7.3 (9.1)	7.3 (9.1)		8.1 (14.5)	8.3 (14.8)	5.2 (6.1)	
Median [IQR]	5 [3–8]	5 [3–8]	5 [3–8]		5 [3–8]	5 [4–9]	4 [3–5]	
ICU admission; n (%) out of patients with acute hospitalization^c^	73 881 (38.4%)	70 375 (38.7%)	3506 (33.5%)	<.001	885 (34.2%)	852 (34.7%)	33 (25.0%)	.029
Use of antiemetics, antidiarrheal agents, and antibiotics within 3 d on or after index date; n (%)								
Overall use	434 326 (25.47%)	340 090 (24.20%)	94 236 (31.36%)	<.001	1886 (32.49%)	1525 (29.94%)	361 (50.70%)	<.001
Use of antiemetics	259 299 (15.20%)	193 498 (13.77%)	65 801 (21.90%)	<.001	1352 (23.29%)	1024 (20.11%)	328 (46.07%)	<.001
Use of antidiarrheal agents	53 488 (3.14%)	47 335 (3.37%)	6153 (2.05%)	<.001	198 (3.41%)	172 (3.38%)	26 (3.65%)	.789
Use of antibiotics	201 150 (11.79%)	162 914 (11.59%)	38 236 (12.73%)	<.001	782 (13.47%)	696 (13.67%)	86 (12.08%)	.270

Abbreviations: AGE, acute gastroenteritis; ICU, intensive care unit; IQR, interquartile range; SD, standard deviation.

^a^Comparisons between individuals with and without underlying medical conditions within each cohort were conducted using the Mann–Whitney *U* test for continuous variables and the χ^2^ test or Fisher's exact test for categorical variables.

^b^Index date is the first day of an episode of all-cause AGE or norovirus AGE, where all eligibility requirements are met. Unless otherwise specified, all percentages are calculated using the total number of eligible episodes as the denominator.

^c^LOS and ICU admission are described among episodes with an acute hospitalization.

Among episodes with acute hospitalization, the median LOS was longer for norovirus AGE episodes with underlying conditions compared with without (5 days [IQR, 4–9] vs 4 days [3–5], respectively; *P* < .001). ICU admission was more frequent for those with underlying conditions in both the all-cause AGE cohort (38.7% vs 33.5%; *P* < .001) and norovirus AGE cohort (34.7% vs 25.0%; *P* = .029). Similar patterns were observed within strata of patients aged 18–64 years and ≥65 years (Supplementary Table 2).

### Associations Between Underlying Medical Conditions and Acute Hospitalization Following All-cause AGE and Norovirus AGE Episodes

Associations between specific underlying conditions and risk of acute hospitalization after all-cause AGE and norovirus AGE are presented in Table 3. Of the conditions assessed, CVD was associated with the highest relative risk of acute hospitalization after both all-cause AGE and norovirus AGE episodes, adjusting for other underlying conditions, age, sex, and region (all-cause AGE: aRR, 1.67 [95% CI, 1.64–1.69]; norovirus AGE: 1.37 [1.22–1.54]). Other conditions associated with an elevated risk of acute hospitalization following AGE episode included blood disorders, chronic respiratory disease, and CKD. Episodes of either all-cause AGE or norovirus AGE with any underlying condition were associated with over twice the risk for acute hospitalization compared with episodes without conditions (all-cause AGE: aRR, 2.52 [95% CI, 2.47–2.58]; norovirus AGE: 2.02 [1.71–2.39]). While episodes with 1 and ≥2 underlying conditions showed increased risk of acute hospitalization compared with the reference group of those without underlying conditions, the magnitude of risk was greater for episodes with ≥2 underlying conditions (all-cause AGE: 2.02 [95% CI, 1.98–2.07]; norovirus: 1.79 [1.50–2.14]) than episodes with 1 underlying condition (all-cause AGE: 1.30 [1.27–1.34]; norovirus AGE: 1.31 [1.07–1.59]). Similarly, when compared with episodes with a CCI of 0, those with a CCI of ≥2 were associated with a higher relative risk of acute hospitalization (all-cause AGE: aRR, 2.25 [95% CI, 2.22–2.29]; norovirus AGE: 1.71 [1.53–1.91]) than those with a CCI of 1 (all-cause AGE: aRR, 1.59 [1.57–1.62]; norovirus AGE: 1.48 [1.30–1.68]).

**Table 3.** Crude and aRRs for Acute Hospitalization Within 3 Days on or After Episodes for All-cause AGE and Cause-specified Norovirus AGE

Exposure	All-cause AGE^a^		Norovirus AGE^b^	
	Crude Risk Ratio (95% CI)	aRR (95% CI)	Crude Risk Ratio (95% CI)	aRR (95% CI)
Model 1: Individual underlying medical conditions (Referent: not having a specific underlying condition)^c^				
Blood disorders	2.67 (2.63–2.71)	1.44 (1.42–1.46)	1.57 (1.44–1.70)	1.19 (1.09–1.29)
CVD	3.46 (3.42–3.51)	1.67 (1.64–1.69)	2.12 (1.92–2.34)	1.37 (1.22–1.54)
Chronic respiratory disease	2.35 (2.33–2.37)	1.49 (1.48–1.51)	1.50 (1.42–1.60)	1.17 (1.10–1.25)
Chronic liver disease	1.96 (1.93–1.98)	1.34 (1.33–1.36)	1.44 (1.32–1.57)	1.23 (1.13–1.33)
CKD	2.60 (2.57–2.62)	1.45 (1.44–1.47)	1.57 (1.48–1.67)	1.16 (1.09–1.24)
Diabetes (types I and II)	1.85 (1.84–1.87)	1.14 (1.13–1.15)	1.42 (1.34–1.50)	1.12 (1.05–1.19)
Obesity	1.49 (1.47–1.50)	1.09 (1.08–1.10)	1.21 (1.13–1.29)	1.00 (0.93–1.07)
Neurological disorders	2.43 (2.40–2.45)	1.43 (1.41–1.44)	1.43 (1.35–1.52)	1.09 (1.02–1.16)
Chronic gastrointestinal disease	1.64 (1.62–1.65)	1.05 (1.04–1.06)	1.28 (1.21–1.36)	1.01 (0.94–1.07)
Chronic immunocompromising conditions	1.61 (1.59–1.63)	1.13 (1.12–1.14)	1.36 (1.27–1.45)	1.16 (1.09–1.24)
Model 2: Any underlying medical condition (Referent: no study-defined underlying conditions)^d^				
Any underlying medical condition	3.71 (3.64–3.79)	2.52 (2.47–2.58)	2.58 (2.21–3.02)	2.02 (1.71–2.39)
Model 3: Number of underlying medical conditions (Referent: no study-defined underlying conditions)^e^				
Categorical number of underlying conditions				
1 condition	1.55 (1.51–1.59)	1.30 (1.27–1.34)	1.51 (1.24–1.83)	1.31 (1.07–1.59)
≥2 conditions	2.73 (2.67–2.79)	2.02 (1.98–2.07)	2.22 (1.88–2.62)	1.79 (1.50–2.14)
Model 4: Charlson-Quan comorbidity index (Referent: CCI score of 0)^f^				
Categorical Charlson-Quan comorbidity index				
Score of 1	1.83 (1.80–1.86)	1.59 (1.57–1.62)	1.63 (1.43–1.85)	1.48 (1.30–1.68)
Score of ≥2	2.78 (2.74–2.82)	2.25 (2.22–2.29)	1.95 (1.76–2.17)	1.71 (1.53–1.91)

Abbreviations: AGE, acute gastroenteritis; CCI, Charlson-Quan comorbidity index; CI, confidence interval; GEE, generalized estimating equation.

^a^A small proportion of all-cause AGE episodes (0.22%, n = 3801) were excluded for having unknown or missing sex information, or other, unknown, or missing region information, resulting in 1 701 713 AGE episodes available for analysis in the GEE models.

^b^A small proportion of norovirus AGE episodes (0.16%, n = 9) were excluded, resulting in 5796 episodes for analysis.

^c^Model 1: log(E[Acute hospitalization]) = β0 + β1*Blood disorders + β2*Cardiovascular disease + β3*Obesity + β4*Diabetes + β5*Chronic kidney disease + β6*Neurologic disorder + β7*Immunocompromised + β8*Chronic respiratory disease + β9*Chronic liver disease + β10*Chronic gastrointestinal disease + β11*Age + β12*Sex + β13*Region.

^d^Model 2: log(E[Acute hospitalization]) = β0 + β1*Any underlying medical condition + β2*Age + β3*Sex + β4*Region.

^e^Model 3: log(E[Acute hospitalization]) = β0 + β1*Number of underlying conditions + β2*Age + β3*Sex + β4*Region.

^f^Model 4: log(E[Acute hospitalization]) = β0 + β1*Charlson-Quan comorbidity index + β2*Age + β3*Sex + β4*Region.

**Figure 2.**
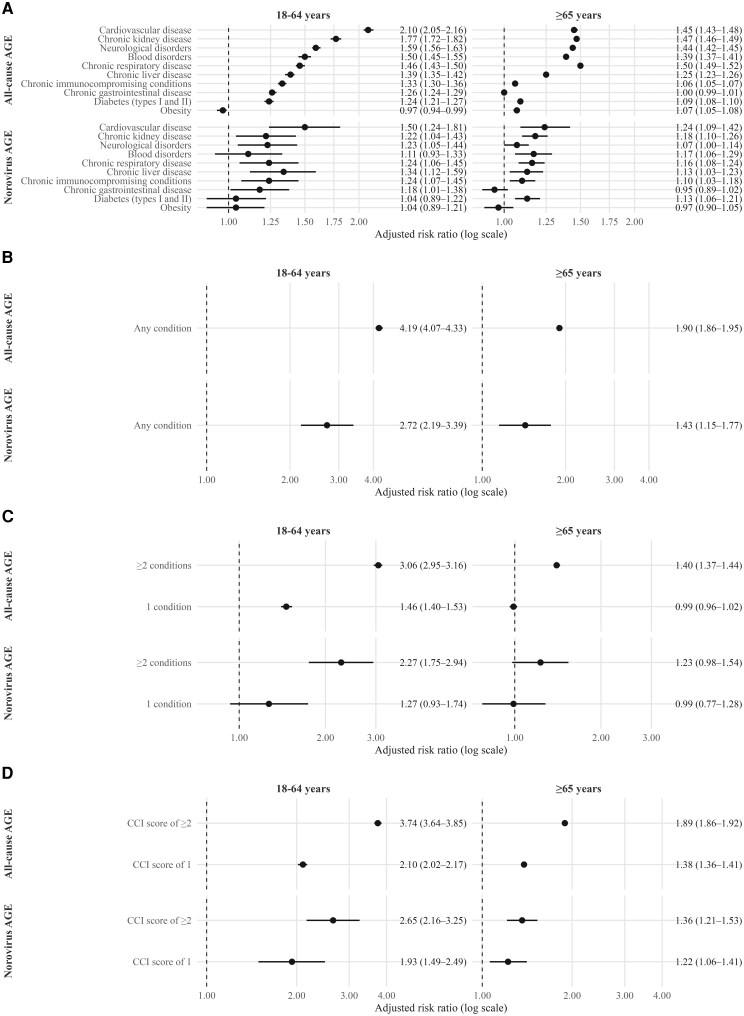
Panels *A–D* illustrate the relative risk (with 95% CIs) of acute hospitalization, adjusted for sex and US Census region in GEE models, for all-cause AGE and norovirus AGE episodes within age-stratified groups (18–64 and ≥65 y). *A*, Relative risk of hospitalization varied for each underlying medical condition, adjusting for other conditions (referent: not having a specific underlying condition). *B*, Relative risk among patients with at least one underlying condition was elevated for the younger age group (referent: no study-defined underlying conditions). *C*, Relative risk increased with number of conditions (referent: no study-defined underlying conditions) and (*D*) with CCI (referent: CCI score of 0). Abbreviations: AGE, acute gastroenteritis; CCI, Charlson-Quan comorbidity index; GEE, generalized estimating equation.

In age-stratified GEE models (Figure 2), episodes from adults aged 18–64 years with chronic gastrointestinal disease were associated with a higher risk of acute hospitalization compared with episodes from those without the condition (all-cause AGE: aRR, 1.26 [95% CI, 1.24–1.29]; norovirus AGE: 1.18 [1.01–1.38]), but chronic gastrointestinal disease did not appear to increase the risk among adults aged ≥65 years (all-cause AGE: 1.00 [0.99–1.01]; norovirus AGE: 0.95 [0.89–1.02]) (Figure 2*A*). Episodes with underlying conditions compared with the reference group of those without underlying conditions were associated with a greater relative risk of acute hospitalization among adults aged 18–64 years (all-cause AGE: aRR, 4.19 [95% CI, 4.07–4.33]; norovirus AGE: 2.72 [2.19–3.39]) than for adults ≥65 years (all-cause AGE: 1.90 [1.86–1.95]; norovirus AGE: 1.43 [1.15–1.77]) (Figure 2*B*). Among adults aged 18–64 years with all-cause AGE, the risk of acute hospitalization increased with the number of underlying conditions compared with the reference group of those without underlying conditions (Figure 2*C*). Among adults aged ≥65 years, no significant difference was observed between those with 1 condition and no underlying condition. Compared with the reference group with a CCI of 0, the risk of acute hospitalization increased with higher CCI scores for the all-cause AGE cohort, whereas in the norovirus AGE cohort, the relative risk of those with a CCI of 1 did not differ significantly from those with a CCI of ≥2 (Figure 2*D*). Similar patterns were observed among patients aged ≥80 years (Supplementary Table 3); however, the presence of a single underlying condition was not associated with a significantly higher hospitalization risk compared with patients without underlying conditions.

## DISCUSSION

In this real-world cohort study of all-cause AGE or norovirus AGE episodes, the presence of at least one underlying medical condition, notably CVD and multiple underlying conditions, increased the risk of acute hospitalization. While we observed a higher absolute risk of acute hospitalization among adults aged ≥65 years compared with younger adults 18–64 years in both cohorts, the increased risk of hospitalization associated with underlying conditions was greater among younger adults than older adults. Together, findings from this analysis provide new insights characterizing the effects of age and underlying conditions on the risk of acute hospitalization among AGE episodes.

In both the all-cause AGE and cause-specified norovirus AGE cohorts, the presence of any underlying condition was independently associated with an increased risk of acute hospitalization after adjusting for potential confounders, including age, sex, and region. Previous studies have similarly reported associations between chronic illnesses and adverse outcomes of norovirus infection such as death and duration of diarrhea [10, 11].

To date, evidence and guidance have primarily focused on the link between immunocompromised status and severe norovirus illness; current Infectious Diseases Society of America guidelines identify individuals with immunocompromising disorders as a high-risk population warranting diagnostic testing [13, 14]. However, our analysis demonstrated that CVD was also associated with a high relative risk of acute hospitalization. Several potential pathophysiological explanations have been proposed for such an association, including that gastroenteritis may trigger acute myocardial infarctions through inflammatory processes and dehydration, which predispose patients to elevated blood viscosity, venous thromboembolism, and a hypercoagulable state [15]. CVD has also been reported as a risk factor for substantial (>20%) reductions in potassium levels and increased levels of C-reactive protein and creatinine phosphokinase during norovirus infection, potentially contributing to complications for CVD as well as for other conditions with an elevated relative risk in our analysis [11]. Furthermore, medications commonly used to treat CVD, such as diuretics and beta-blockers, may destabilize a patient's physiological response to dehydrating AGE [16]. While these pathophysiological hypotheses are compelling, it is possible that patients presenting with CVD and other underlying conditions are more likely to be hospitalized regardless of AGE, highlighting a need for future research to differentiate between these 2 possibilities.

Multimorbidity was associated with a higher relative risk of acute hospitalization compared with having a single underlying condition. Previous studies characterizing norovirus-related hospitalizations have focused on frail, multimorbid populations such as residents of nursing homes [9], and inpatient healthcare costs among patients with norovirus have been shown to increase with higher CCI [17]. However, there is limited data on the incremental risk of hospitalization associated with having multiple underlying conditions versus a single condition, as well as the risk in younger adults. One descriptive study noted higher hospitalization rates with increased comorbidity indices among the broader AGE population, but this finding was not specific to norovirus [18]. Additionally, Verstraeten et al reported higher hospitalization rates among adults with at least 2 chronic conditions [6]; however, these estimates were age-standardized rate ratios rather than aRRs accounting for other confounders. Our results extend existing evidence by quantifying the additive effect of multiple underlying conditions on hospitalization risk in the norovirus AGE population, suggesting that future studies should explore its implications for clinical management. Future studies may also consider evaluating the Elixhauser Comorbidity Index, which has demonstrated improved performance over CCI in predicting mortality outcomes in large administrative datasets [19].

For both all-cause AGE and norovirus AGE, the proportion of adults with acute hospitalization increased with age. This pattern aligns with prior evidence demonstrating that older adults are at higher risk for severe outcomes of norovirus, including death and hospitalization [4, 5]. Our study reveals that, within age strata, the relative impact of underlying conditions on hospitalization risk was greater among younger adults than among older adults. The attenuated relative risk among older adults likely reflects their higher background hospitalization risk and widespread multimorbidity, which may mask the incremental effect of individual conditions. In younger adults, underlying conditions are often underdiagnosed until they manifest as severe illness, potentially leading to a detection bias where diagnosed individuals skew toward a more vulnerable population. Channeling bias may also contribute to differences in relative risk between the age groups, as older adults are more likely to be admitted regardless of health status, whereas the presence of underlying conditions may be a primary driver for hospitalization among younger adults. In addition, age-related differences in the effects of specific underlying conditions may contribute to the higher relative risk observed among younger adults. Chronic gastrointestinal disease, associated with an increased risk of acute hospitalization in younger but not older adults in this study, may influence risk through age-related differences in gut microbiome composition [20]. Given the role of microbiome profile in norovirus prognosis [21, 22], further investigation of these age-specific mechanisms is warranted. Altogether, these findings highlight the importance of targeted monitoring and management strategies for at-risk adults across the age spectrum, not only among the elderly.

In this study, we were limited to diagnosis codes as diagnostic testing for individuals with AGE is not commonly conducted in routine clinical care, particularly among adults [8, 14]. Thus, the number of norovirus AGE episodes identified is an underestimate of the true burden [23]. While cause-unspecified AGE codes may represent undiagnosed norovirus, we chose to examine cause-specified cases only to isolate those we could be reasonably confident were caused by norovirus, though the BioFire® FilmArray® Gastrointestinal panel's January 2024 recall for false-positive norovirus results may have misclassified some norovirus cases during part of our study period [24]. Among patients with cause-specified norovirus AGE in our analysis, almost half of individuals with norovirus episodes experiencing hospitalization within 3 days of presenting with AGE. Patients with norovirus AGE were also older and had a higher baseline comorbidity burden, suggesting a more clinically complex population compared with patients presenting with all-cause AGE.

A key strength of this study is that it leveraged a geographically diverse, nationally representative US administrative claims dataset to assess a large population of patients with all-cause AGE and norovirus AGE. The 2022–2024 study period gives a more accurate, up-to-date landscape of current risk factors for hospitalization among patients with all-cause AGE and norovirus AGE in a post-COVID-19 period. Additionally, evaluating a broader all-cause AGE population alongside the norovirus AGE population allowed for the inclusion of potentially undiagnosed or misclassified norovirus cases. Consistent patterns across the 2 cohorts strengthened the robustness of the findings.

Findings from this study are subject to the following limitations. First, this study did not exclude all-cause AGE or norovirus AGE episodes in which the initial diagnosis occurred during an inpatient encounter, given the objective was to characterize all-cause AGE and norovirus AGE as they are diagnosed and managed in real-world settings. This approach may overestimate the relative risk of acute hospitalization among patients with norovirus AGE when compared with the all-cause AGE population, as diagnostic testing for norovirus is more frequently performed on patients with critical illness, which drives care-seeking behavior, specimen collection, and eventual hospital admission [14, 25]. The analysis did not apply a washout period for hospitalizations occurring prior to the index date, which could have also contributed to high observed hospitalization risk in both cohorts as these may not have been incident. Present-on-admission designations and other indicators of nosocomial infection were not consistently captured in Optum® CDM, limiting reliable exclusion of nosocomial AGE cases and potentially inflating hospitalization risk estimates. Concurrent enteric diagnoses may have contributed to hospitalization risk among norovirus AGE episodes. In an exploratory assessment, 6% of norovirus AGE episodes had a concurrent *Clostridioides difficile* diagnosis during the episode, similar to the 9% of test-positive norovirus cases previously reported [3]. Because claims data do not reliably distinguish true coinfection from colonization, diagnostic uncertainty, or inpatient testing patterns, these episodes were retained in the primary analysis. Because risk ratios were used, patients disenrolling shortly after their index date could not be accounted for, though the 3-day outcome window made censoring minimal and unlikely to affect results. As is common with real-world data, information on social determinants of health was limited. Race and ethnicity were excluded from adjusted models due to ∼20% missing data, and Optum® CDM lacks geographic detail to derive rurality or area deprivation index. Finally, this analysis utilized administrative claims data from US commercial insurance and Medicare Advantage records and focused on individuals with at least 12 months of continuous insurance coverage. As a result, the findings may not be generalizable to the broader US population, particularly individuals without healthcare insurance or with other types of insurance (eg, Medicaid, Medicare Fee-for-Service), or to the global population.

In conclusion, this study demonstrated that underlying medical conditions, particularly CVD and multimorbidity, substantially increased the risk of hospitalization on or shortly after encounters for all-cause AGE and norovirus AGE. Blood disorders, chronic respiratory disease, and acute kidney disease were also strongly associated with an increased risk of hospitalization. Although older adults exhibited a higher absolute risk of hospitalization than younger adults, the latter group experienced a greater relative increase in risk when underlying conditions were present, providing evidence suggesting increased burden in younger adults with underlying conditions. Future research should further investigate how age, multimorbidity, and specific underlying conditions such as CVD interact to influence hospitalization risk, using comparisons with reference populations without AGE to clarify whether these associations are unique to norovirus infection or reflect broader patterns of susceptibility to infections.

## Supplementary Material

ofag449_Supplementary_Data

## Supplementary Data

Supplementary materials are available at *Open Forum Infectious Diseases* online. Consisting of data provided by the authors to benefit the reader, the posted materials are not copyedited and are the sole responsibility of the authors, so questions or comments should be addressed to the corresponding author.
