# Supplementary material for: Individual-level Factors Associated With Acute Hospitalization After Medically Attended Acute Gastroenteritis and Norovirus Gastroenteritis in the United States, 2022–2024
Source: Open Forum Infect Dis. 2026 Jul 27;13(7):ofag449. doi: 10.1093/ofid/ofag449 (PMC13420534; doi:10.1093/ofid/ofag449)
Supplement: ofag449_Supplementary_Data [file ofag449_supplementary_data.docx]

# Supplementary Data

**Supplementary Table 1.** Code list for study variables

| **Variable** | **Definition** |
| --- | --- |
| All-cause acute gastroenteritis | *Including codes for norovirus, rotavirus, adenovirus, unspecified viral enteritis, Clostridium difficile and other bacterial intestinal disease, parasitic intestinal disease, and unspecified diarrhea.*  **ICD-10-CM:**  A00, A00.0, A00.1, A00.9, A01, A01.0, A01.00, A01.01, A01.02, A01.03, A01.04, A01.05, A01.09, A01.1, A01.2, A01.3, A01.4, A02, A02.0, A02.1, A02.2, A02.20, A02.21, A02.22, A02.23, A02.24, A02.25, A02.29, A02.8, A02.9, A03, A03.0, A03.1, A03.2, A03.3, A03.8, A03.9, A04, A04.0, A04.1, A04.2, A04.3, A04.4, A04.5, A04.6, A04.7, A04.71, A04.72, A04.8, A04.9, A05, A05.0, A05.1, A05.2, A05.3, A05.4, A05.5, A05.8, A05.9, A06, A06.0, A06.1, A06.2, A06.3, A06.8, A06.81, A06.82, A06.89, A06.9, A07, A07.0, A07.1, A07.2, A07.3, A07.8, A07.9, A08.0, A08.1, A08.11, A08.19, A08.2, A08.3, A08.31, A08.32, A08.39, A08.4, A08.5, A08.8, A09, A09.0, A09.9, K52.8, K52.81, K52.82, K52.83, K52.831, K52.832, K52.838, K52.839, K52.89, K52.9, R19.7 |
| Norovirus acute gastroenteritis | **ICD-10-CM:**  A08.1, A08.11, A08.19 |
| Blood disorders | *Including but not limited to codes for unspecified anemia, thalassemia, sickle cell disorders, hereditary hemolytic anemias, and coagulation defects.*  **ICD-10-CM:**  D55, D55.0, D55.1, D55.2, D55.21, D55.29, D55.3, D55.8, D55.9, D56.0, D56.1, D56.2, D56.4, D56.5, D56.8, D56.9, D57.0, D57.00, D57.01, D57.02, D57.03, D57.04, D57.09, D57.1, D57.2, D57.20, D57.21, D57.211, D57.212, D57.213, D57.214, D57.218, D57.219, D57.4, D57.40, D57.41, D57.411, D57.412, D57.413, D57.414, D57.418, D57.419, D57.42, D57.43, D57.431, D57.432, D57.433, D57.434, D57.438, D57.439, D57.44, D57.45, D57.451, D57.452, D57.453, D57.454, D57.458, D57.459, D57.8, D57.80, D57.81, D57.811, D57.812, D57.813, D57.814, D57.818, D57.819, D58, D58.0, D58.1, D58.2, D58.8, D58.9, D64.0, D64.1, D64.2, D64.3, D64.4, D64.8, D64.81, D64.89, D65, D66, D67, D68, D68.0, D68.00, D68.01, D68.02, D68.020, D68.021, D68.022, D68.023, D68.029, D68.03, D68.04, D68.09, D68.1, D68.2, D68.3, D68.31, D68.311, D68.312, D68.318, D68.32, D68.4, D68.5, D68.51, D68.52, D68.59, D68.6, D68.61, D68.62, D68.69, D68.8, D68.9 |
| Cardiovascular disease | *Including but not limited to codes for cardiovascular and cerebrovascular syphilis, rheumatic heart disease, hypertensive heart disease, angina pectoris, acute myocardial infarction, chronic and acute ischemic heart disease, pulmonary heart disease, acute pericarditis, acute endocarditis, nonrheumatic valve disorder, cardiac arrest, atrial fibrillation, heart failure, atherosclerosis, aneurysm, atheroembolism, and congenital malformations of cardiac septa,*  **ICD-10-CM:**  A52.0, A52.00, A52.01, A52.02, A52.03, A52.04, A52.05, A52.06, A52.09, B37.6, I01, I01.0, I01.1, I01.2, I01.8, I01.9, I05, I05.0, I05.1, I05.2, I05.8, I05.9, I06, I06.0, I06.1, I06.2, I06.8, I06.9, I07, I07.0, I07.1, I07.2, I07.8, I07.9, I08, I08.0, I08.1, I08.2, I08.3, I08.8, I08.9, I09, I09.0, I09.1, I09.2, I09.8, I09.81, I09.89, I09.9, I10, I11, I11.0, I11.9, I13.0, I15, I15.0, I15.1, I15.2, I15.8, I15.9, I20, I20.0, I20.1, I20.2, I20.8, I20.81, I20.89, I20.9, I21, I21.0, I21.01, I21.02, I21.09, I21.1, I21.11, I21.19, I21.2, I21.21, I21.29, I21.3, I21.4, I21.9, I21.A, I21.A1, I21.A9, I21.B, I22, I22.0, I22.1, I22.2, I22.8, I22.9, I23, I23.0, I23.1, I23.2, I23.3, I23.4, I23.5, I23.6, I23.7, I23.8, I24, I24.0, I24.1, I24.8, I24.81, I24.89, I24.9, I25, I25.1, I25.10, I25.11, I25.110, I25.111, I25.112, I25.118, I25.119, I25.2, I25.3, I25.4, I25.41, I25.42, I25.5, I25.6, I25.7, I25.70, I25.700, I25.701, I25.702, I25.708, I25.709, I25.71, I25.710, I25.711, I25.712, I25.718, I25.719, I25.72, I25.720, I25.721, I25.722, I25.728, I25.729, I25.73, I25.730, I25.731, I25.732, I25.738, I25.739, I25.75, I25.750, I25.751, I25.752, I25.758, I25.759, I25.76, I25.760, I25.761, I25.762, I25.768, I25.769, I25.79, I25.790, I25.791, I25.792, I25.798, I25.799, I25.8, I25.81, I25.810, I25.811, I25.812, I25.82, I25.83, I25.84, I25.85, I25.89, I25.9, I26, I26.0, I26.01, I26.02, I26.03, I26.04, I26.09, I26.9, I26.90, I26.92, I26.93, I26.94, I26.95, I26.96, I26.99, I27, I27.0, I27.1, I27.2, I27.20, I27.21, I27.22, I27.23, I27.24, I27.29, I27.8, I27.81, I27.82, I27.83, I27.89, I27.9, I28, I28.0, I28.1, I28.8, I28.9, I30, I30.0, I30.1, I30.8, I30.9, I31, I31.0, I31.1, I31.2, I31.3, I31.31, I31.39, I31.4, I31.8, I31.9, I32, I33, I33.0, I33.9, I34, I34.0, I34.1, I34.2, I34.8, I34.81, I34.89, I34.9, I35, I35.0, I35.1, I35.2, I35.8, I35.9, I36, I36.0, I36.1, I36.2, I36.8, I36.9, I37, I37.0, I37.1, I37.2, I37.8, I37.9, I38, I39, I40, I40.0, I40.1, I40.8, I40.9, I41, I42, I42.0, I42.1, I42.2, I42.3, I42.4, I42.5, I42.6, I42.7, I42.8, I42.9, I43, I44, I44.0, I44.1, I44.2, I44.3, I44.30, I44.39, I44.4, I44.5, I44.6, I44.60, I44.69, I44.7, I45, I45.0, I45.1, I45.10, I45.19, I45.2, I45.3, I45.4, I45.5, I45.6, I45.8, I45.81, I45.89, I45.9, I46, I46.2, I46.8, I46.9, I48, I48.0, I48.1, I48.11, I48.19, I48.2, I48.20, I48.21, I48.3, I48.4, I48.9, I48.91, I48.92, I49.01, I49.02, I49.1, I49.2, I49.3, I49.4, I49.40, I49.49, I49.5, I49.8, I49.9, I50, I50.1, I50.2, I50.20, I50.21, I50.22, I50.23, I50.3, I50.30, I50.31, I50.32, I50.33, I50.4, I50.40, I50.41, I50.42, I50.43, I50.8, I50.81, I50.810, I50.811, I50.812, I50.813, I50.814, I50.82, I50.83, I50.84, I50.89, I50.9, I51, I51.0, I51.1, I51.2, I51.3, I51.4, I51.5, I51.7, I51.8, I51.81, I51.89, I51.9, I52, I5A, I70, I70.0, I70.1, I70.2, I70.20, I70.201, I70.202, I70.203, I70.208, I70.209, I70.21, I70.211, I70.212, I70.213, I70.218, I70.219, I70.22, I70.221, I70.222, I70.223, I70.228, I70.229, I70.23, I70.231, I70.232, I70.233, I70.234, I70.235, I70.238, I70.239, I70.24, I70.241, I70.242, I70.243, I70.244, I70.245, I70.248, I70.249, I70.25, I70.26, I70.261, I70.262, I70.263, I70.268, I70.269, I70.29, I70.291, I70.292, I70.293, I70.298, I70.299, I70.3, I70.30, I70.301, I70.302, I70.303, I70.308, I70.309, I70.31, I70.311, I70.312, I70.313, I70.318, I70.319, I70.32, I70.321, I70.322, I70.323, I70.328, I70.329, I70.33, I70.331, I70.332, I70.333, I70.334, I70.335, I70.338, I70.339, I70.34, I70.341, I70.342, I70.343, I70.344, I70.345, I70.348, I70.349, I70.35, I70.36, I70.361, I70.362, I70.363, I70.368, I70.369, I70.39, I70.391, I70.392, I70.393, I70.398, I70.399, I70.4, I70.40, I70.401, I70.402, I70.403, I70.408, I70.409, I70.41, I70.411, I70.412, I70.413, I70.418, I70.419, I70.42, I70.421, I70.422, I70.423, I70.428, I70.429, I70.43, I70.431, I70.432, I70.433, I70.434, I70.435, I70.438, I70.439, I70.44, I70.441, I70.442, I70.443, I70.444, I70.445, I70.448, I70.449, I70.45, I70.46, I70.461, I70.462, I70.463, I70.468, I70.469, I70.49, I70.491, I70.492, I70.493, I70.498, I70.499, I70.5, I70.50, I70.501, I70.502, I70.503, I70.508, I70.509, I70.51, I70.511, I70.512, I70.513, I70.518, I70.519, I70.52, I70.521, I70.522, I70.523, I70.528, I70.529, I70.53, I70.531, I70.532, I70.533, I70.534, I70.535, I70.538, I70.539, I70.54, I70.541, I70.542, I70.543, I70.544, I70.545, I70.548, I70.549, I70.55, I70.56, I70.561, I70.562, I70.563, I70.568, I70.569, I70.59, I70.591, I70.592, I70.593, I70.598, I70.599, I70.6, I70.60, I70.601, I70.602, I70.603, I70.608, I70.609, I70.61, I70.611, I70.612, I70.613, I70.618, I70.619, I70.62, I70.621, I70.622, I70.623, I70.628, I70.629, I70.63, I70.631, I70.632, I70.633, I70.634, I70.635, I70.638, I70.639, I70.64, I70.641, I70.642, I70.643, I70.644, I70.645, I70.648, I70.649, I70.65, I70.66, I70.661, I70.662, I70.663, I70.668, I70.669, I70.69, I70.691, I70.692, I70.693, I70.698, I70.699, I70.7, I70.70, I70.701, I70.702, I70.703, I70.708, I70.709, I70.71, I70.711, I70.712, I70.713, I70.718, I70.719, I70.72, I70.721, I70.722, I70.723, I70.728, I70.729, I70.73, I70.731, I70.732, I70.733, I70.734, I70.735, I70.738, I70.739, I70.74, I70.741, I70.742, I70.743, I70.744, I70.745, I70.748, I70.749, I70.75, I70.76, I70.761, I70.762, I70.763, I70.768, I70.769, I70.79, I70.791, I70.792, I70.793, I70.798, I70.799, I70.8, I70.9, I70.90, I70.91, I70.92, I71, I71.0, I71.00, I71.01, I71.010, I71.011, I71.012, I71.019, I71.02, I71.03, I71.1, I71.10, I71.11, I71.12, I71.13, I71.2, I71.20, I71.21, I71.22, I71.23, I71.3, I71.30, I71.31, I71.32, I71.33, I71.4, I71.40, I71.41, I71.42, I71.43, I71.5, I71.50, I71.51, I71.52, I71.6, I71.60, I71.61, I71.62, I71.8, I71.9, I72, I72.0, I72.1, I72.2, I72.3, I72.4, I72.5, I72.6, I72.8, I72.9, I73, I73.0, I73.00, I73.01, I73.1, I73.8, I73.81, I73.89, I73.9, I74, I74.0, I74.01, I74.09, I74.1, I74.10, I74.11, I74.19, I74.2, I74.3, I74.4, I74.5, I74.8, I74.9, I75, I75.0, I75.01, I75.011, I75.012, I75.013, I75.019, I75.02, I75.021, I75.022, I75.023, I75.029, I75.8, I75.81, I75.89, I76, I77, I77.0, I77.1, I77.2, I77.3, I77.4, I77.5, I77.6, I77.7, I77.70, I77.71, I77.72, I77.73, I77.74, I77.75, I77.76, I77.77, I77.79, I77.8, I77.81, I77.810, I77.811, I77.812, I77.819, I77.82, I77.89, I77.9, Q20, Q20.0, Q20.1, Q20.2, Q20.3, Q20.4, Q20.5, Q20.6, Q20.8, Q20.9, Q21, Q21.0, Q21.1, Q21.10, Q21.11, Q21.12, Q21.13, Q21.14, Q21.15, Q21.16, Q21.19, Q21.2, Q21.20, Q21.21, Q21.22, Q21.23, Q21.3, Q21.4, Q21.8, Q21.9, Q22, Q22.0, Q22.1, Q22.2, Q22.3, Q22.4, Q22.5, Q22.6, Q22.8, Q22.9, Q23, Q23.0, Q23.1, Q23.2, Q23.3, Q23.4, Q23.8, Q23.81, Q23.82, Q23.88, Q23.9, Q24, Q24.0, Q24.1, Q24.2, Q24.3, Q24.4, Q24.5, Q24.6, Q24.8, Q24.9, Q25, Q25.0, Q25.1, Q25.2, Q25.21, Q25.29, Q25.3, Q25.4, Q25.40, Q25.41, Q25.42, Q25.43, Q25.44, Q25.45, Q25.46, Q25.47, Q25.48, Q25.49, Q25.5, Q25.6, Q25.7, Q25.71, Q25.72, Q25.79, Q25.8, Q25.9, Q26, Q26.0, Q26.1, Q26.2, Q26.3, Q26.4, Q26.5, Q26.6, Q26.8, Q26.9, Q27, Q27.0, Q27.1, Q27.2, Q27.3, Q27.30, Q27.31, Q27.32, Q27.33, Q27.34, Q27.39, Q27.4, Q27.8, Q27.9, Q28, Q28.0, Q28.1, Q28.2, Q28.3, Q28.8, Q28.9 |
| Chronic gastrointestinal disease | *Including but not limited to codes for ulcer, esophageal obstruction, gastritis, duodenitis, disease of stomach and duodenum, Chrohn’s disease, ulcerative colitis, inflammatory polyps of colon, unspecified noninfective gastroenteritis and colitis, ileus, diverticular disease of intestine, irritable bowel syndrome, anorectal fistula, disease of anus and rectum, hemorrhoids, cholelithiasis, and intestinal malabsorption.*  **ICD-10-CM:**  K22.1, K22.10, K22.11, K22.2, K22.5, K22.7, K22.70, K22.71, K22.710, K22.711, K22.719, K23.0, K23.1, K25, K25.0, K25.1, K25.2, K25.3, K25.4, K25.5, K25.6, K25.7, K25.9, K26, K26.0, K26.1, K26.2, K26.3, K26.4, K26.5, K26.6, K26.7, K26.9, K27, K27.0, K27.1, K27.2, K27.3, K27.4, K27.5, K27.6, K27.7, K27.9, K28, K28.0, K28.1, K28.2, K28.3, K28.4, K28.5, K28.6, K28.7, K28.9, K29.2, K29.20, K29.21, K29.3, K29.30, K29.31, K29.4, K29.40, K29.41, K29.5, K29.50, K29.51, K29.6, K29.60, K29.61, K29.7, K29.70, K29.71, K29.8, K29.80, K29.81, K29.9, K29.90, K29.91, K31.1, K31.2, K31.3, K31.4, K31.5, K31.6, K31.7, K31.8, K31.81, K31.811, K31.819, K31.82, K31.83, K31.84, K31.89, K31.9, K50, K50.0, K50.00, K50.01, K50.011, K50.012, K50.013, K50.014, K50.018, K50.019, K50.1, K50.10, K50.11, K50.111, K50.112, K50.113, K50.114, K50.118, K50.119, K50.8, K50.80, K50.81, K50.811, K50.812, K50.813, K50.814, K50.818, K50.819, K50.9, K50.90, K50.91, K50.911, K50.912, K50.913, K50.914, K50.918, K50.919, K51, K51.0, K51.00, K51.01, K51.011, K51.012, K51.013, K51.014, K51.018, K51.019, K51.2, K51.20, K51.21, K51.211, K51.212, K51.213, K51.214, K51.218, K51.219, K51.3, K51.30, K51.31, K51.311, K51.312, K51.313, K51.314, K51.318, K51.319, K51.4, K51.40, K51.41, K51.411, K51.412, K51.413, K51.414, K51.418, K51.419, K51.5, K51.50, K51.51, K51.511, K51.512, K51.513, K51.514, K51.518, K51.519, K51.8, K51.80, K51.81, K51.811, K51.812, K51.813, K51.814, K51.818, K51.819, K51.9, K51.90, K51.91, K51.911, K51.912, K51.913, K51.914, K51.918, K51.919, K52, K52.0, K52.1, K52.2, K52.21, K52.22, K52.29, K52.3, K52.8, K52.81, K52.82, K52.83, K52.831, K52.832, K52.838, K52.839, K52.89, K52.9, K55, K55.0, K55.01, K55.011, K55.012, K55.019, K55.02, K55.021, K55.022, K55.029, K55.03, K55.031, K55.032, K55.039, K55.04, K55.041, K55.042, K55.049, K55.05, K55.051, K55.052, K55.059, K55.06, K55.061, K55.062, K55.069, K55.1, K55.2, K55.20, K55.21, K55.3, K55.30, K55.31, K55.32, K55.33, K55.8, K55.9, K56.0, K56.3, K56.5, K56.50, K56.51, K56.52, K56.6, K56.60, K56.600, K56.601, K56.609, K56.69, K56.690, K56.691, K56.699, K56.7, K57, K57.0, K57.00, K57.01, K57.1, K57.10, K57.11, K57.12, K57.13, K57.2, K57.20, K57.21, K57.3, K57.30, K57.31, K57.32, K57.33, K57.4, K57.40, K57.41, K57.5, K57.50, K57.51, K57.52, K57.53, K57.8, K57.80, K57.81, K57.9, K57.90, K57.91, K57.92, K57.93, K58, K58.0, K58.1, K58.2, K58.8, K58.9, K59, K59.0, K59.00, K59.01, K59.02, K59.03, K59.04, K59.09, K59.1, K59.2, K59.3, K59.31, K59.39, K59.4, K59.8, K59.81, K59.89, K59.9, K60.1, K60.5, K60.50, K60.51, K60.511, K60.512, K60.513, K60.519, K60.52, K60.521, K60.522, K60.523, K60.529, K62, K62.0, K62.1, K62.2, K62.3, K62.4, K62.5, K62.6, K62.7, K62.8, K62.81, K62.82, K62.89, K62.9, K63, K63.0, K63.1, K63.2, K63.3, K63.4, K63.5, K63.8, K63.81, K63.82, K63.821, K63.8211, K63.8212, K63.8219, K63.822, K63.829, K63.89, K63.9, K64, K64.0, K64.1, K64.2, K64.3, K64.4, K64.5, K64.8, K64.9, K66, K66.0, K66.1, K66.8, K66.9, K67, K80, K80.0, K80.00, K80.01, K80.1, K80.10, K80.11, K80.12, K80.13, K80.18, K80.19, K80.2, K80.20, K80.21, K80.3, K80.30, K80.31, K80.32, K80.33, K80.34, K80.35, K80.36, K80.37, K80.4, K80.40, K80.41, K80.42, K80.43, K80.44, K80.45, K80.46, K80.47, K80.5, K80.50, K80.51, K80.6, K80.60, K80.61, K80.62, K80.63, K80.64, K80.65, K80.66, K80.67, K80.7, K80.70, K80.71, K80.8, K80.80, K80.81, K81.1, K82, K82.0, K82.1, K82.2, K82.3, K82.4, K82.8, K82.9, K82.A, K82.A1, K82.A2, K83, K83.0, K83.01, K83.09, K83.1, K83.2, K83.3, K83.4, K83.5, K83.8, K83.9, K86, K86.0, K86.1, K86.2, K86.3, K86.8, K86.81, K86.89, K86.9, K87, K90, K90.0, K90.1, K90.2, K90.3, K90.4, K90.41, K90.49, K90.8, K90.81, K90.82, K90.821, K90.822, K90.829, K90.83, K90.89, K90.9, K92, K92.0, K92.1, K92.2, K92.8, K92.81, K92.89, K92.9, K93, K93.0, K93.1, K93.8, K94.0, K94.00, K94.01, K94.02, K94.03, K94.09, K94.1, K94.10, K94.11, K94.12, K94.13, K94.19, Q38, Q38.0, Q38.1, Q38.2, Q38.3, Q38.4, Q38.5, Q38.6, Q38.7, Q38.8, Q39, Q39.0, Q39.1, Q39.2, Q39.3, Q39.4, Q39.5, Q39.6, Q39.8, Q39.9, Q40, Q40.0, Q40.1, Q40.2, Q40.3, Q40.8, Q40.9, Q41, Q41.0, Q41.1, Q41.2, Q41.8, Q41.9, Q42, Q42.0, Q42.1, Q42.2, Q42.3, Q42.8, Q42.9, Q43, Q43.0, Q43.1, Q43.2, Q43.3, Q43.4, Q43.5, Q43.6, Q43.7, Q43.8, Q43.9, Q44, Q44.0, Q44.1, Q44.2, Q44.3, Q44.4, Q44.5, Q44.6, Q44.7, Q44.70, Q44.71, Q44.79, Q45, Q45.0, Q45.1, Q45.2, Q45.3, Q45.8, Q45.9  *K50* and K51* additionally require the non-occurrence of immunotherapy/chemotherapy in the baseline period.* |
| Chronic kidney disease | *Including but not limited to codes for hypertensive chronic kidney disease, nephritic syndrome, chronic kidney disease (all stages), and renal dialysis.*  **ICD-10-CM:**  I12, I12.0, I12.9, I13.1, I13.10, I13.11, I13.2, N03, N03.0, N03.1, N03.2, N03.3, N03.4, N03.5, N03.6, N03.7, N03.8, N03.9, N03.A, N04, N04.0, N04.1, N04.2, N04.20, N04.21, N04.22, N04.29, N04.3, N04.4, N04.5, N04.6, N04.7, N04.8, N04.9, N04.A, N05, N05.0, N05.1, N05.2, N05.3, N05.4, N05.5, N05.6, N05.7, N05.8, N05.9, N05.A, N18, N18.1, N18.2, N18.3, N18.30, N18.31, N18.32, N18.4, N18.5, N18.6, N18.9, N19, N25, N25.0, N25.1, N25.8, N25.81, N25.89, N25.9, R80.9, Z49.0, Z49.01, Z49.02, Z49.1, Z49.2, Z49.3, Z49.31, Z49.32, Z91.15, Z91.151, Z91.158, Z99.2  **CPT:**  90935, 90937, 90945, 90947  **Revenue codes:**  0800, 0801, 0802, 0803, 0804, 0809, 0820, 0821, 0822, 0823, 0824, 0825, 0829, 0830, 0831, 0832, 0833, 0834, 0835, 0839, 0840, 0841, 0842, 0843, 0844, 0845, 0849, 0850, 0851, 0852, 0853, 0854, 0855, 0859, 0880, 0881, 0882, 0889 |
| Chronic liver disease | *Including but not limited to codes for viral hepatitis, esophageal varices, alcoholic liver disease, toxic liver disease, hepatic failure, hepatitis, and hepatic fibrosis.*  **ICD-10-CM:**  B18, B18.0, B18.1, B18.2, B18.8, B18.9, B19, B19.0, B19.1, B19.10, B19.11, B19.2, B19.20, B19.21, B19.9, I85, I85.0, I85.00, I85.01, I85.1, I85.10, I85.11, I86.4, I98.2, K70, K70.0, K70.1, K70.10, K70.11, K70.2, K70.3, K70.30, K70.31, K70.4, K70.40, K70.41, K70.9, K71.1, K71.10, K71.11, K71.3, K71.4, K71.5, K71.50, K71.51, K71.6, K71.7, K71.8, K71.9, K72, K72.0, K72.00, K72.01, K72.1, K72.10, K72.11, K72.9, K72.90, K72.91, K73, K73.0, K73.1, K73.2, K73.8, K73.9, K74, K74.0, K74.00, K74.01, K74.02, K74.1, K74.2, K74.3, K74.4, K74.5, K74.6, K74.60, K74.69, K75.2, K75.3, K75.4, K75.8, K75.81, K75.89, K75.9, K76, K76.0, K76.1, K76.2, K76.3, K76.4, K76.5, K76.6, K76.7, K76.8, K76.81, K76.82, K76.89, K76.9 |
| Chronic respiratory disease | *Including but not limited to codes for tuberculosis, cystic fibrosis, rhinitis, sinusitis, nasal polyp, chronic disease of tonsils and adenoids, chronic laryngitis, disease of upper respiratory tract, bronchitis, emphysema, chronic obstructive pulmonary disease, asthma, bronchiectasis, pneumoconiosis, pneumonia, pulmonary fibrosis, pyothorax, pleural effusion, and respiratory failure.*  **ICD-10-CM:**  A15, A15.0, A15.4, A15.5, A15.6, A15.7, A15.8, A15.9, A16, A16.0, A16.1, A16.2, A16.3, A16.4, A16.5, A16.7, A16.8, A16.9, A19, A19.0, A19.1, A19.2, A19.8, A19.9, A31.0, E84, E84.0, E84.1, E84.11, E84.19, E84.8, E84.9, I27.8, I27.81, I27.82, I27.83, I27.89, J30, J30.0, J30.1, J30.2, J30.5, J30.8, J30.81, J30.89, J30.9, J32, J32.0, J32.1, J32.2, J32.3, J32.4, J32.8, J32.9, J33, J33.0, J33.1, J33.8, J33.9, J34, J34.0, J34.1, J34.2, J34.3, J34.8, J34.81, J34.82, J34.820, J34.8200, J34.8201, J34.8202, J34.821, J34.8210, J34.8211, J34.8212, J34.829, J34.89, J34.9, J35, J35.0, J35.01, J35.02, J35.03, J35.1, J35.2, J35.3, J35.8, J35.9, J36, J37, J37.0, J37.1, J38, J38.0, J38.00, J38.01, J38.02, J38.1, J38.2, J38.3, J38.4, J38.5, J38.6, J38.7, J39, J39.0, J39.1, J39.2, J39.3, J39.8, J39.9, J40, J41, J41.0, J41.1, J41.8, J42, J43, J43.0, J43.1, J43.2, J43.8, J43.9, J44, J44.0, J44.1, J44.8, J44.81, J44.89, J44.9, J45, J45.2, J45.20, J45.21, J45.22, J45.3, J45.30, J45.31, J45.32, J45.4, J45.40, J45.41, J45.42, J45.5, J45.50, J45.51, J45.52, J45.9, J45.90, J45.901, J45.902, J45.909, J45.99, J45.990, J45.991, J45.998, J47, J47.0, J47.1, J47.9, J4A, J4A.0, J4A.8, J4A.9, J60, J61, J62, J62.0, J62.8, J63, J63.0, J63.1, J63.2, J63.3, J63.4, J63.5, J63.6, J64, J65, J66, J66.0, J66.1, J66.2, J66.8, J67, J67.0, J67.1, J67.2, J67.3, J67.4, J67.5, J67.6, J67.7, J67.8, J67.9, J68, J68.0, J68.1, J68.2, J68.3, J68.4, J68.8, J68.9, J69, J69.0, J69.1, J69.8, J70.0, J70.1, J70.3, J70.4, J70.5, J70.8, J70.9, J81.1, J82, J82.8, J82.81, J82.82, J82.83, J82.89, J84, J84.0, J84.01, J84.02, J84.03, J84.09, J84.1, J84.10, J84.11, J84.111, J84.112, J84.113, J84.114, J84.115, J84.116, J84.117, J84.17, J84.170, J84.178, J84.2, J84.8, J84.81, J84.82, J84.83, J84.84, J84.841, J84.842, J84.843, J84.848, J84.89, J84.9, J85, J85.0, J85.1, J85.2, J85.3, J86, J86.0, J86.9, J90, J91, J91.0, J91.8, J92, J92.0, J92.9, J93, J93.0, J93.1, J93.11, J93.12, J93.8, J93.81, J93.82, J93.83, J93.9, J94, J94.0, J94.1, J94.2, J94.8, J94.9, J95, J95.0, J95.00, J95.01, J95.02, J95.03, J95.04, J95.09, J95.1, J95.2, J95.3, J95.4, J95.5, J95.6, J95.61, J95.62, J95.7, J95.71, J95.72, J95.8, J95.81, J95.811, J95.812, J95.82, J95.821, J95.822, J95.83, J95.830, J95.831, J95.84, J95.85, J95.850, J95.851, J95.859, J95.86, J95.860, J95.861, J95.862, J95.863, J95.87, J95.88, J95.89, J96, J96.0, J96.00, J96.01, J96.02, J96.1, J96.10, J96.11, J96.12, J96.2, J96.20, J96.21, J96.22, J96.9, J96.90, J96.91, J96.92, J98, J98.0, J98.01, J98.09, J98.1, J98.11, J98.19, J98.2, J98.3, J98.4, J98.5, J98.51, J98.59, J98.6, J98.8, J98.9, J99, P09.4, Q32, Q32.0, Q32.1, Q32.2, Q32.3, Q32.4, Q33, Q33.0, Q33.1, Q33.2, Q33.3, Q33.4, Q33.5, Q33.6, Q33.8, Q33.9, Z14.1 |
| Diabetes | *Including codes for type 1 diabetes mellitus, type 2 diabetes mellitus, and other specified diabetes mellitus.*  **ICD-10-CM:**  E10, E10.1, E10.10, E10.11, E10.2, E10.21, E10.22, E10.29, E10.3, E10.31, E10.311, E10.319, E10.32, E10.321, E10.3211, E10.3212, E10.3213, E10.3219, E10.329, E10.3291, E10.3292, E10.3293, E10.3299, E10.33, E10.331, E10.3311, E10.3312, E10.3313, E10.3319, E10.339, E10.3391, E10.3392, E10.3393, E10.3399, E10.34, E10.341, E10.3411, E10.3412, E10.3413, E10.3419, E10.349, E10.3491, E10.3492, E10.3493, E10.3499, E10.35, E10.351, E10.3511, E10.3512, E10.3513, E10.3519, E10.352, E10.3521, E10.3522, E10.3523, E10.3529, E10.353, E10.3531, E10.3532, E10.3533, E10.3539, E10.354, E10.3541, E10.3542, E10.3543, E10.3549, E10.355, E10.3551, E10.3552, E10.3553, E10.3559, E10.359, E10.3591, E10.3592, E10.3593, E10.3599, E10.36, E10.37, E10.37X1, E10.37X2, E10.37X3, E10.37X9, E10.39, E10.4, E10.40, E10.41, E10.42, E10.43, E10.44, E10.49, E10.5, E10.51, E10.52, E10.59, E10.6, E10.61, E10.610, E10.618, E10.62, E10.620, E10.621, E10.622, E10.628, E10.63, E10.630, E10.638, E10.64, E10.641, E10.649, E10.65, E10.69, E10.8, E10.9, E10.A, E10.A0, E10.A1, E10.A2, E11, E11.0, E11.00, E11.01, E11.1, E11.10, E11.11, E11.2, E11.21, E11.22, E11.29, E11.3, E11.31, E11.311, E11.319, E11.32, E11.321, E11.3211, E11.3212, E11.3213, E11.3219, E11.329, E11.3291, E11.3292, E11.3293, E11.3299, E11.33, E11.331, E11.3311, E11.3312, E11.3313, E11.3319, E11.339, E11.3391, E11.3392, E11.3393, E11.3399, E11.34, E11.341, E11.3411, E11.3412, E11.3413, E11.3419, E11.349, E11.3491, E11.3492, E11.3493, E11.3499, E11.35, E11.351, E11.3511, E11.3512, E11.3513, E11.3519, E11.352, E11.3521, E11.3522, E11.3523, E11.3529, E11.353, E11.3531, E11.3532, E11.3533, E11.3539, E11.354, E11.3541, E11.3542, E11.3543, E11.3549, E11.355, E11.3551, E11.3552, E11.3553, E11.3559, E11.359, E11.3591, E11.3592, E11.3593, E11.3599, E11.36, E11.37, E11.37X1, E11.37X2, E11.37X3, E11.37X9, E11.39, E11.4, E11.40, E11.41, E11.42, E11.43, E11.44, E11.49, E11.5, E11.51, E11.52, E11.59, E11.6, E11.61, E11.610, E11.618, E11.62, E11.620, E11.621, E11.622, E11.628, E11.63, E11.630, E11.638, E11.64, E11.641, E11.649, E11.65, E11.69, E11.8, E11.9, E13, E13.0, E13.00, E13.01, E13.1, E13.10, E13.11, E13.2, E13.21, E13.22, E13.29, E13.3, E13.31, E13.311, E13.319, E13.32, E13.321, E13.3211, E13.3212, E13.3213, E13.3219, E13.329, E13.3291, E13.3292, E13.3293, E13.3299, E13.33, E13.331, E13.3311, E13.3312, E13.3313, E13.3319, E13.339, E13.3391, E13.3392, E13.3393, E13.3399, E13.34, E13.341, E13.3411, E13.3412, E13.3413, E13.3419, E13.349, E13.3491, E13.3492, E13.3493, E13.3499, E13.35, E13.351, E13.3511, E13.3512, E13.3513, E13.3519, E13.352, E13.3521, E13.3522, E13.3523, E13.3529, E13.353, E13.3531, E13.3532, E13.3533, E13.3539, E13.354, E13.3541, E13.3542, E13.3543, E13.3549, E13.355, E13.3551, E13.3552, E13.3553, E13.3559, E13.359, E13.3591, E13.3592, E13.3593, E13.3599, E13.36, E13.37, E13.37X1, E13.37X2, E13.37X3, E13.37X9, E13.39, E13.4, E13.40, E13.41, E13.42, E13.43, E13.44, E13.49, E13.5, E13.51, E13.52, E13.59, E13.6, E13.61, E13.610, E13.618, E13.62, E13.620, E13.621, E13.622, E13.628, E13.63, E13.630, E13.638, E13.64, E13.641, E13.649, E13.65, E13.69, E13.8, E13.9 |
| Immunocompromising disorders | *Including but not limited to codes for mycobacterial infection, human immunodeficiency virus disease, mycosis, toxoplasmosis, malignant neoplasm, malignant melanoma, carcinoma, mesothelioma, sarcoma, lymphoma, leukemia, immunodeficiency, sarcoidosis, Crohn's disease or ulcerative colitis with active immunotherapy treatment, psoriasis, rheumatoid arthritis, juvenile arthritis, systemic sclerosis, inflammatory spondylopathy, osteomyelitis, and organ and tissue transplant.*  **ICD-10-CM:**  Codes that require the occurrence of immunotherapy/chemotherapy in the baseline period:  C00, C00.0, C00.1, C00.2, C00.3, C00.4, C00.5, C00.6, C00.8, C00.9, C01, C02, C02.0, C02.1, C02.2, C02.3, C02.4, C02.8, C02.9, C03, C03.0, C03.1, C03.9, C04, C04.0, C04.1, C04.8, C04.9, C05, C05.0, C05.1, C05.2, C05.8, C05.9, C06, C06.0, C06.1, C06.2, C06.8, C06.80, C06.89, C06.9, C07, C08, C08.0, C08.1, C08.9, C09, C09.0, C09.1, C09.8, C09.9, C10, C10.0, C10.1, C10.2, C10.3, C10.4, C10.8, C10.9, C11, C11.0, C11.1, C11.2, C11.3, C11.8, C11.9, C12, C13, C13.0, C13.1, C13.2, C13.8, C13.9, C14, C14.0, C14.2, C14.8, C15, C15.3, C15.4, C15.5, C15.8, C15.9, C16, C16.0, C16.1, C16.2, C16.3, C16.4, C16.5, C16.6, C16.8, C16.9, C17, C17.0, C17.1, C17.2, C17.3, C17.8, C17.9, C18, C18.0, C18.1, C18.2, C18.3, C18.4, C18.5, C18.6, C18.7, C18.8, C18.9, C19, C20, C21, C21.0, C21.1, C21.2, C21.8, C22, C22.0, C22.1, C22.2, C22.3, C22.4, C22.7, C22.8, C22.9, C23, C24, C24.0, C24.1, C24.8, C24.9, C25, C25.0, C25.1, C25.2, C25.3, C25.4, C25.7, C25.8, C25.9, C26, C26.0, C26.1, C26.9, C30, C30.0, C30.1, C31, C31.0, C31.1, C31.2, C31.3, C31.8, C31.9, C32, C32.0, C32.1, C32.2, C32.3, C32.8, C32.9, C33, C34, C34.0, C34.00, C34.01, C34.02, C34.1, C34.10, C34.11, C34.12, C34.2, C34.3, C34.30, C34.31, C34.32, C34.8, C34.80, C34.81, C34.82, C34.9, C34.90, C34.91, C34.92, C37, C38, C38.0, C38.1, C38.2, C38.3, C38.4, C38.8, C39, C39.0, C39.9, C40, C40.0, C40.00, C40.01, C40.02, C40.1, C40.10, C40.11, C40.12, C40.2, C40.20, C40.21, C40.22, C40.3, C40.30, C40.31, C40.32, C40.8, C40.80, C40.81, C40.82, C40.9, C40.90, C40.91, C40.92, C41, C41.0, C41.1, C41.2, C41.3, C41.4, C41.9, C43, C43.0, C43.1, C43.10, C43.11, C43.111, C43.112, C43.12, C43.121, C43.122, C43.2, C43.20, C43.21, C43.22, C43.3, C43.30, C43.31, C43.39, C43.4, C43.5, C43.51, C43.52, C43.59, C43.6, C43.60, C43.61, C43.62, C43.7, C43.70, C43.71, C43.72, C43.8, C43.9, C44, C44.0, C44.00, C44.01, C44.02, C44.09, C44.1, C44.10, C44.101, C44.102, C44.1021, C44.1022, C44.109, C44.1091, C44.1092, C44.11, C44.111, C44.112, C44.1121, C44.1122, C44.119, C44.1191, C44.1192, C44.12, C44.121, C44.122, C44.1221, C44.1222, C44.129, C44.1291, C44.1292, C44.13, C44.131, C44.132, C44.1321, C44.1322, C44.139, C44.1391, C44.1392, C44.19, C44.191, C44.192, C44.1921, C44.1922, C44.199, C44.1991, C44.1992, C44.2, C44.20, C44.201, C44.202, C44.209, C44.21, C44.211, C44.212, C44.219, C44.22, C44.221, C44.222, C44.229, C44.29, C44.291, C44.292, C44.299, C44.3, C44.30, C44.300, C44.301, C44.309, C44.31, C44.310, C44.311, C44.319, C44.32, C44.320, C44.321, C44.329, C44.39, C44.390, C44.391, C44.399, C44.4, C44.40, C44.41, C44.42, C44.49, C44.5, C44.50, C44.500, C44.501, C44.509, C44.51, C44.510, C44.511, C44.519, C44.52, C44.520, C44.521, C44.529, C44.59, C44.590, C44.591, C44.599, C44.6, C44.60, C44.601, C44.602, C44.609, C44.61, C44.611, C44.612, C44.619, C44.62, C44.621, C44.622, C44.629, C44.69, C44.691, C44.692, C44.699, C44.7, C44.70, C44.701, C44.702, C44.709, C44.71, C44.711, C44.712, C44.719, C44.72, C44.721, C44.722, C44.729, C44.79, C44.791, C44.792, C44.799, C44.8, C44.80, C44.81, C44.82, C44.89, C44.9, C44.90, C44.91, C44.92, C44.99, C45, C45.0, C45.1, C45.2, C45.7, C45.9, C46, C46.0, C46.1, C46.2, C46.3, C46.4, C46.5, C46.50, C46.51, C46.52, C46.7, C46.9, C47, C47.0, C47.1, C47.10, C47.11, C47.12, C47.2, C47.20, C47.21, C47.22, C47.3, C47.4, C47.5, C47.6, C47.8, C47.9, C48, C48.0, C48.1, C48.2, C48.8, C49, C49.0, C49.1, C49.10, C49.11, C49.12, C49.2, C49.20, C49.21, C49.22, C49.3, C49.4, C49.5, C49.6, C49.8, C49.9, C49.A, C49.A0, C49.A1, C49.A2, C49.A3, C49.A4, C49.A5, C49.A9, C4A, C4A.0, C4A.1, C4A.10, C4A.11, C4A.111, C4A.112, C4A.12, C4A.121, C4A.122, C4A.2, C4A.20, C4A.21, C4A.22, C4A.3, C4A.30, C4A.31, C4A.39, C4A.4, C4A.5, C4A.51, C4A.52, C4A.59, C4A.6, C4A.60, C4A.61, C4A.62, C4A.7, C4A.70, C4A.71, C4A.72, C4A.8, C4A.9, C50, C50.0, C50.01, C50.011, C50.012, C50.019, C50.02, C50.021, C50.022, C50.029, C50.1, C50.11, C50.111, C50.112, C50.119, C50.12, C50.121, C50.122, C50.129, C50.2, C50.21, C50.211, C50.212, C50.219, C50.22, C50.221, C50.222, C50.229, C50.3, C50.31, C50.311, C50.312, C50.319, C50.32, C50.321, C50.322, C50.329, C50.4, C50.41, C50.411, C50.412, C50.419, C50.42, C50.421, C50.422, C50.429, C50.5, C50.51, C50.511, C50.512, C50.519, C50.52, C50.521, C50.522, C50.529, C50.6, C50.61, C50.611, C50.612, C50.619, C50.62, C50.621, C50.622, C50.629, C50.8, C50.81, C50.811, C50.812, C50.819, C50.82, C50.821, C50.822, C50.829, C50.9, C50.91, C50.911, C50.912, C50.919, C50.92, C50.921, C50.922, C50.929, C51, C51.0, C51.1, C51.2, C51.8, C51.9, C52, C53, C53.0, C53.1, C53.8, C53.9, C54, C54.0, C54.1, C54.2, C54.3, C54.8, C54.9, C55, C56, C56.1, C56.2, C56.3, C56.9, C57, C57.0, C57.00, C57.01, C57.02, C57.1, C57.10, C57.11, C57.12, C57.2, C57.20, C57.21, C57.22, C57.3, C57.4, C57.7, C57.8, C57.9, C58, C60, C60.0, C60.1, C60.2, C60.8, C60.9, C61, C62, C62.0, C62.00, C62.01, C62.02, C62.1, C62.10, C62.11, C62.12, C62.9, C62.90, C62.91, C62.92, C63, C63.0, C63.00, C63.01, C63.02, C63.1, C63.10, C63.11, C63.12, C63.2, C63.7, C63.8, C63.9, C64, C64.1, C64.2, C64.9, C65, C65.1, C65.2, C65.9, C66, C66.1, C66.2, C66.9, C67, C67.0, C67.1, C67.2, C67.3, C67.4, C67.5, C67.6, C67.7, C67.8, C67.9, C68, C68.0, C68.1, C68.8, C68.9, C69, C69.0, C69.00, C69.01, C69.02, C69.1, C69.10, C69.11, C69.12, C69.2, C69.20, C69.21, C69.22, C69.3, C69.30, C69.31, C69.32, C69.4, C69.40, C69.41, C69.42, C69.5, C69.50, C69.51, C69.52, C69.6, C69.60, C69.61, C69.62, C69.8, C69.80, C69.81, C69.82, C69.9, C69.90, C69.91, C69.92, C70, C70.0, C70.1, C70.9, C71, C71.0, C71.1, C71.2, C71.3, C71.4, C71.5, C71.6, C71.7, C71.8, C71.9, C72, C72.0, C72.1, C72.2, C72.20, C72.21, C72.22, C72.3, C72.30, C72.31, C72.32, C72.4, C72.40, C72.41, C72.42, C72.5, C72.50, C72.59, C72.9, C73, C74, C74.0, C74.00, C74.01, C74.02, C74.1, C74.10, C74.11, C74.12, C74.9, C74.90, C74.91, C74.92, C75, C75.0, C75.1, C75.2, C75.3, C75.4, C75.5, C75.8, C75.9, C76, C76.0, C76.1, C76.2, C76.3, C76.4, C76.40, C76.41, C76.42, C76.5, C76.50, C76.51, C76.52, C76.8, C77, C77.0, C77.1, C77.2, C77.3, C77.4, C77.5, C77.8, C77.9, C78, C78.0, C78.00, C78.01, C78.02, C78.1, C78.2, C78.3, C78.30, C78.39, C78.4, C78.5, C78.6, C78.7, C78.8, C78.80, C78.89, C79, C79.0, C79.00, C79.01, C79.02, C79.1, C79.10, C79.11, C79.19, C79.2, C79.3, C79.31, C79.32, C79.4, C79.40, C79.49, C79.5, C79.51, C79.52, C79.6, C79.60, C79.61, C79.62, C79.63, C79.7, C79.70, C79.71, C79.72, C79.8, C79.81, C79.82, C79.89, C79.9, C7A, C7A.0, C7A.00, C7A.01, C7A.010, C7A.011, C7A.012, C7A.019, C7A.02, C7A.020, C7A.021, C7A.022, C7A.023, C7A.024, C7A.025, C7A.026, C7A.029, C7A.09, C7A.090, C7A.091, C7A.092, C7A.093, C7A.094, C7A.095, C7A.096, C7A.098, C7A.1, C7A.8, C7B, C7B.0, C7B.00, C7B.01, C7B.02, C7B.03, C7B.04, C7B.09, C7B.1, C7B.8, C80, C80.0, C80.1, C80.2, D37, D37.0, D37.01, D37.02, D37.03, D37.030, D37.031, D37.032, D37.039, D37.04, D37.05, D37.09, D37.1, D37.2, D37.3, D37.4, D37.5, D37.6, D37.8, D37.9, D38, D38.0, D38.1, D38.2, D38.3, D38.4, D38.5, D38.6, D39, D39.0, D39.1, D39.10, D39.11, D39.12, D39.2, D39.8, D39.9, D40, D40.0, D40.1, D40.10, D40.11, D40.12, D40.8, D40.9, D41, D41.0, D41.00, D41.01, D41.02, D41.1, D41.10, D41.11, D41.12, D41.2, D41.20, D41.21, D41.22, D41.3, D41.4, D41.8, D41.9, D42, D42.0, D42.1, D42.9, D43, D43.0, D43.1, D43.2, D43.3, D43.4, D43.8, D43.9, D44, D44.0, D44.1, D44.10, D44.11, D44.12, D44.2, D44.3, D44.4, D44.5, D44.6, D44.7, D44.9, D45, D46, D46.0, D46.1, D46.2, D46.20, D46.21, D46.22, D46.4, D46.9, D46.A, D46.B, D46.C, D46.Z, D47, D47.0, D47.01, D47.02, D47.09, D47.1, D47.2, D47.3, D47.4, D47.9, D47.Z, D47.Z2, D47.Z9, D48, D48.0, D48.1, D48.11, D48.110, D48.111, D48.112, D48.113, D48.114, D48.115, D48.116, D48.117, D48.118, D48.119, D48.19, D48.2, D48.3, D48.4, D48.5, D48.6, D48.60, D48.61, D48.62, D48.7, D48.9, D68.312, D68.62, D86, D86.0, D86.1, D86.2, D86.3, D86.8, D86.81, D86.82, D86.83, D86.84, D86.85, D86.86, D86.87, D86.89, D86.9, G35, K50, K50.0, K50.00, K50.01, K50.011, K50.012, K50.013, K50.014, K50.018, K50.019, K50.1, K50.10, K50.11, K50.111, K50.112, K50.113, K50.114, K50.118, K50.119, K50.8, K50.80, K50.81, K50.811, K50.812, K50.813, K50.814, K50.818, K50.819, K50.9, K50.90, K50.91, K50.911, K50.912, K50.913, K50.914, K50.918, K50.919, K51, K51.0, K51.00, K51.01, K51.011, K51.012, K51.013, K51.014, K51.018, K51.019, K51.2, K51.20, K51.21, K51.211, K51.212, K51.213, K51.214, K51.218, K51.219, K51.3, K51.30, K51.31, K51.311, K51.312, K51.313, K51.314, K51.318, K51.319, K51.4, K51.40, K51.41, K51.411, K51.412, K51.413, K51.414, K51.418, K51.419, K51.5, K51.50, K51.51, K51.511, K51.512, K51.513, K51.514, K51.518, K51.519, K51.8, K51.80, K51.81, K51.811, K51.812, K51.813, K51.814, K51.818, K51.819, K51.9, K51.90, K51.91, K51.911, K51.912, K51.913, K51.914, K51.918, K51.919, L40, L40.0, L40.1, L40.2, L40.3, L40.4, L40.5, L40.50, L40.51, L40.52, L40.53, L40.54, L40.59, L40.8, L40.9, M05, M05.0, M05.00, M05.01, M05.011, M05.012, M05.019, M05.02, M05.021, M05.022, M05.029, M05.03, M05.031, M05.032, M05.039, M05.04, M05.041, M05.042, M05.049, M05.05, M05.051, M05.052, M05.059, M05.06, M05.061, M05.062, M05.069, M05.07, M05.071, M05.072, M05.079, M05.09, M05.1, M05.10, M05.11, M05.111, M05.112, M05.119, M05.12, M05.121, M05.122, M05.129, M05.13, M05.131, M05.132, M05.139, M05.14, M05.141, M05.142, M05.149, M05.15, M05.151, M05.152, M05.159, M05.16, M05.161, M05.162, M05.169, M05.17, M05.171, M05.172, M05.179, M05.19, M05.2, M05.20, M05.21, M05.211, M05.212, M05.219, M05.22, M05.221, M05.222, M05.229, M05.23, M05.231, M05.232, M05.239, M05.24, M05.241, M05.242, M05.249, M05.25, M05.251, M05.252, M05.259, M05.26, M05.261, M05.262, M05.269, M05.27, M05.271, M05.272, M05.279, M05.29, M05.3, M05.30, M05.31, M05.311, M05.312, M05.319, M05.32, M05.321, M05.322, M05.329, M05.33, M05.331, M05.332, M05.339, M05.34, M05.341, M05.342, M05.349, M05.35, M05.351, M05.352, M05.359, M05.36, M05.361, M05.362, M05.369, M05.37, M05.371, M05.372, M05.379, M05.39, M05.4, M05.40, M05.41, M05.411, M05.412, M05.419, M05.42, M05.421, M05.422, M05.429, M05.43, M05.431, M05.432, M05.439, M05.44, M05.441, M05.442, M05.449, M05.45, M05.451, M05.452, M05.459, M05.46, M05.461, M05.462, M05.469, M05.47, M05.471, M05.472, M05.479, M05.49, M05.5, M05.50, M05.51, M05.511, M05.512, M05.519, M05.52, M05.521, M05.522, M05.529, M05.53, M05.531, M05.532, M05.539, M05.54, M05.541, M05.542, M05.549, M05.55, M05.551, M05.552, M05.559, M05.56, M05.561, M05.562, M05.569, M05.57, M05.571, M05.572, M05.579, M05.59, M05.6, M05.60, M05.61, M05.611, M05.612, M05.619, M05.62, M05.621, M05.622, M05.629, M05.63, M05.631, M05.632, M05.639, M05.64, M05.641, M05.642, M05.649, M05.65, M05.651, M05.652, M05.659, M05.66, M05.661, M05.662, M05.669, M05.67, M05.671, M05.672, M05.679, M05.69, M05.7, M05.70, M05.71, M05.711, M05.712, M05.719, M05.72, M05.721, M05.722, M05.729, M05.73, M05.731, M05.732, M05.739, M05.74, M05.741, M05.742, M05.749, M05.75, M05.751, M05.752, M05.759, M05.76, M05.761, M05.762, M05.769, M05.77, M05.771, M05.772, M05.779, M05.79, M05.7A, M05.8, M05.80, M05.81, M05.811, M05.812, M05.819, M05.82, M05.821, M05.822, M05.829, M05.83, M05.831, M05.832, M05.839, M05.84, M05.841, M05.842, M05.849, M05.85, M05.851, M05.852, M05.859, M05.86, M05.861, M05.862, M05.869, M05.87, M05.871, M05.872, M05.879, M05.89, M05.8A, M05.9, M06, M06.0, M06.00, M06.01, M06.011, M06.012, M06.019, M06.02, M06.021, M06.022, M06.029, M06.03, M06.031, M06.032, M06.039, M06.04, M06.041, M06.042, M06.049, M06.05, M06.051, M06.052, M06.059, M06.06, M06.061, M06.062, M06.069, M06.07, M06.071, M06.072, M06.079, M06.08, M06.09, M06.0A, M06.1, M06.2, M06.20, M06.21, M06.211, M06.212, M06.219, M06.22, M06.221, M06.222, M06.229, M06.23, M06.231, M06.232, M06.239, M06.24, M06.241, M06.242, M06.249, M06.25, M06.251, M06.252, M06.259, M06.26, M06.261, M06.262, M06.269, M06.27, M06.271, M06.272, M06.279, M06.28, M06.29, M06.3, M06.30, M06.31, M06.311, M06.312, M06.319, M06.32, M06.321, M06.322, M06.329, M06.33, M06.331, M06.332, M06.339, M06.34, M06.341, M06.342, M06.349, M06.35, M06.351, M06.352, M06.359, M06.36, M06.361, M06.362, M06.369, M06.37, M06.371, M06.372, M06.379, M06.38, M06.39, M06.4, M06.8, M06.80, M06.81, M06.811, M06.812, M06.819, M06.82, M06.821, M06.822, M06.829, M06.83, M06.831, M06.832, M06.839, M06.84, M06.841, M06.842, M06.849, M06.85, M06.851, M06.852, M06.859, M06.86, M06.861, M06.862, M06.869, M06.87, M06.871, M06.872, M06.879, M06.88, M06.89, M06.8A, M06.9, M07, M07.6, M07.60, M07.61, M07.611, M07.612, M07.619, M07.62, M07.621, M07.622, M07.629, M07.63, M07.631, M07.632, M07.639, M07.64, M07.641, M07.642, M07.649, M07.65, M07.651, M07.652, M07.659, M07.66, M07.661, M07.662, M07.669, M07.67, M07.671, M07.672, M07.679, M07.68, M07.69, M08, M08.0, M08.00, M08.01, M08.011, M08.012, M08.019, M08.02, M08.021, M08.022, M08.029, M08.03, M08.031, M08.032, M08.039, M08.04, M08.041, M08.042, M08.049, M08.05, M08.051, M08.052, M08.059, M08.06, M08.061, M08.062, M08.069, M08.07, M08.071, M08.072, M08.079, M08.08, M08.09, M08.0A, M08.1, M08.2, M08.20, M08.21, M08.211, M08.212, M08.219, M08.22, M08.221, M08.222, M08.229, M08.23, M08.231, M08.232, M08.239, M08.24, M08.241, M08.242, M08.249, M08.25, M08.251, M08.252, M08.259, M08.26, M08.261, M08.262, M08.269, M08.27, M08.271, M08.272, M08.279, M08.28, M08.29, M08.2A, M08.3, M08.4, M08.40, M08.41, M08.411, M08.412, M08.419, M08.42, M08.421, M08.422, M08.429, M08.43, M08.431, M08.432, M08.439, M08.44, M08.441, M08.442, M08.449, M08.45, M08.451, M08.452, M08.459, M08.46, M08.461, M08.462, M08.469, M08.47, M08.471, M08.472, M08.479, M08.48, M08.4A, M08.8, M08.80, M08.81, M08.811, M08.812, M08.819, M08.82, M08.821, M08.822, M08.829, M08.83, M08.831, M08.832, M08.839, M08.84, M08.841, M08.842, M08.849, M08.85, M08.851, M08.852, M08.859, M08.86, M08.861, M08.862, M08.869, M08.87, M08.871, M08.872, M08.879, M08.88, M08.89, M08.9, M08.90, M08.91, M08.911, M08.912, M08.919, M08.92, M08.921, M08.922, M08.929, M08.93, M08.931, M08.932, M08.939, M08.94, M08.941, M08.942, M08.949, M08.95, M08.951, M08.952, M08.959, M08.96, M08.961, M08.962, M08.969, M08.97, M08.971, M08.972, M08.979, M08.98, M08.99, M08.9A, M30, M30.0, M30.1, M30.2, M30.3, M30.8, M31, M31.0, M31.1, M31.10, M31.11, M31.19, M31.2, M31.3, M31.30, M31.31, M31.4, M31.5, M31.6, M31.7, M31.8, M31.9, M33, M33.0, M33.00, M33.01, M33.02, M33.03, M33.09, M33.1, M33.10, M33.11, M33.12, M33.13, M33.19, M33.2, M33.20, M33.21, M33.22, M33.29, M33.9, M33.90, M33.91, M33.92, M33.93, M33.99, M34, M34.0, M34.1, M34.2, M34.8, M34.81, M34.82, M34.83, M34.89, M34.9, M35.00, M35.01, M35.02, M35.03, M35.04, M35.09, M35.1, M35.2, M35.3, M35.4, M35.5, M35.6, M35.7, M35.8, M35.81, M35.89, M35.9, M36.0, M45, M45.0, M45.1, M45.2, M45.3, M45.4, M45.5, M45.6, M45.7, M45.8, M45.9, M45.A, M45.A0, M45.A1, M45.A2, M45.A3, M45.A4, M45.A5, M45.A6, M45.A7, M45.A8, M45.AB, M46, M46.0, M46.00, M46.01, M46.02, M46.03, M46.04, M46.05, M46.06, M46.07, M46.08, M46.09, M46.1, M46.2, M46.20, M46.21, M46.22, M46.23, M46.24, M46.25, M46.26, M46.27, M46.28, M46.3, M46.30, M46.31, M46.32, M46.33, M46.34, M46.35, M46.36, M46.37, M46.38, M46.39, M46.4, M46.40, M46.41, M46.42, M46.43, M46.44, M46.45, M46.46, M46.47, M46.48, M46.49, M46.5, M46.50, M46.51, M46.52, M46.53, M46.54, M46.55, M46.56, M46.57, M46.58, M46.59, M46.8, M46.80, M46.81, M46.82, M46.83, M46.84, M46.85, M46.86, M46.87, M46.88, M46.89, M46.9, M46.90, M46.91, M46.92, M46.93, M46.94, M46.95, M46.96, M46.97, M46.98, M46.99, Q90, Q90.0, Q90.1, Q90.2, Q90.9, R18.0  Codes that do not require the occurrence of immunotherapy/chemotherapy in the baseline period:  A31, A31.0, A31.1, A31.2, A31.8, A31.9, B20, B23.0, B48, B48.0, B48.1, B48.2, B48.3, B48.4, B48.8, B58, B58.0, B58.00, B58.01, B58.09, B58.1, B58.2, B58.3, B58.8, B58.81, B58.82, B58.83, B58.89, B58.9, B97.35, C81, C81.0, C81.00, C81.01, C81.02, C81.03, C81.04, C81.05, C81.06, C81.07, C81.08, C81.09, C81.0A, C81.1, C81.10, C81.11, C81.12, C81.13, C81.14, C81.15, C81.16, C81.17, C81.18, C81.19, C81.1A, C81.2, C81.20, C81.21, C81.22, C81.23, C81.24, C81.25, C81.26, C81.27, C81.28, C81.29, C81.2A, C81.3, C81.30, C81.31, C81.32, C81.33, C81.34, C81.35, C81.36, C81.37, C81.38, C81.39, C81.3A, C81.4, C81.40, C81.41, C81.42, C81.43, C81.44, C81.45, C81.46, C81.47, C81.48, C81.49, C81.4A, C81.7, C81.70, C81.71, C81.72, C81.73, C81.74, C81.75, C81.76, C81.77, C81.78, C81.79, C81.7A, C81.9, C81.90, C81.91, C81.92, C81.93, C81.94, C81.95, C81.96, C81.97, C81.98, C81.99, C81.9A, C82, C82.0, C82.00, C82.01, C82.02, C82.03, C82.04, C82.05, C82.06, C82.07, C82.08, C82.09, C82.0A, C82.1, C82.10, C82.11, C82.12, C82.13, C82.14, C82.15, C82.16, C82.17, C82.18, C82.19, C82.1A, C82.2, C82.20, C82.21, C82.22, C82.23, C82.24, C82.25, C82.26, C82.27, C82.28, C82.29, C82.2A, C82.3, C82.30, C82.31, C82.32, C82.33, C82.34, C82.35, C82.36, C82.37, C82.38, C82.39, C82.3A, C82.4, C82.40, C82.41, C82.42, C82.43, C82.44, C82.45, C82.46, C82.47, C82.48, C82.49, C82.4A, C82.5, C82.50, C82.51, C82.52, C82.53, C82.54, C82.55, C82.56, C82.57, C82.58, C82.59, C82.5A, C82.6, C82.60, C82.61, C82.62, C82.63, C82.64, C82.65, C82.66, C82.67, C82.68, C82.69, C82.6A, C82.8, C82.80, C82.81, C82.82, C82.83, C82.84, C82.85, C82.86, C82.87, C82.88, C82.89, C82.8A, C82.9, C82.90, C82.91, C82.92, C82.93, C82.94, C82.95, C82.96, C82.97, C82.98, C82.99, C82.9A, C83, C83.0, C83.00, C83.01, C83.02, C83.03, C83.04, C83.05, C83.06, C83.07, C83.08, C83.09, C83.0A, C83.1, C83.10, C83.11, C83.12, C83.13, C83.14, C83.15, C83.16, C83.17, C83.18, C83.19, C83.1A, C83.3, C83.30, C83.31, C83.32, C83.33, C83.34, C83.35, C83.36, C83.37, C83.38, C83.39, C83.390, C83.398, C83.3A, C83.5, C83.50, C83.51, C83.52, C83.53, C83.54, C83.55, C83.56, C83.57, C83.58, C83.59, C83.5A, C83.7, C83.70, C83.71, C83.72, C83.73, C83.74, C83.75, C83.76, C83.77, C83.78, C83.79, C83.7A, C83.8, C83.80, C83.81, C83.82, C83.83, C83.84, C83.85, C83.86, C83.87, C83.88, C83.89, C83.8A, C83.9, C83.90, C83.91, C83.92, C83.93, C83.94, C83.95, C83.96, C83.97, C83.98, C83.99, C83.9A, C84, C84.0, C84.00, C84.01, C84.02, C84.03, C84.04, C84.05, C84.06, C84.07, C84.08, C84.09, C84.0A, C84.1, C84.10, C84.11, C84.12, C84.13, C84.14, C84.15, C84.16, C84.17, C84.18, C84.19, C84.1A, C84.4, C84.40, C84.41, C84.42, C84.43, C84.44, C84.45, C84.46, C84.47, C84.48, C84.49, C84.4A, C84.6, C84.60, C84.61, C84.62, C84.63, C84.64, C84.65, C84.66, C84.67, C84.68, C84.69, C84.6A, C84.7, C84.70, C84.71, C84.72, C84.73, C84.74, C84.75, C84.76, C84.77, C84.78, C84.79, C84.7A, C84.7B, C84.9, C84.90, C84.91, C84.92, C84.93, C84.94, C84.95, C84.96, C84.97, C84.98, C84.99, C84.9A, C84.A, C84.A0, C84.A1, C84.A2, C84.A3, C84.A4, C84.A5, C84.A6, C84.A7, C84.A8, C84.A9, C84.AA, C84.Z, C84.Z0, C84.Z1, C84.Z2, C84.Z3, C84.Z4, C84.Z5, C84.Z6, C84.Z7, C84.Z8, C84.Z9, C84.ZA, C85, C85.1, C85.10, C85.11, C85.12, C85.13, C85.14, C85.15, C85.16, C85.17, C85.18, C85.19, C85.1A, C85.2, C85.20, C85.21, C85.22, C85.23, C85.24, C85.25, C85.26, C85.27, C85.28, C85.29, C85.2A, C85.8, C85.80, C85.81, C85.82, C85.83, C85.84, C85.85, C85.86, C85.87, C85.88, C85.89, C85.8A, C85.9, C85.90, C85.91, C85.92, C85.93, C85.94, C85.95, C85.96, C85.97, C85.98, C85.99, C85.9A, C86, C86.0, C86.00, C86.01, C86.1, C86.10, C86.11, C86.2, C86.20, C86.21, C86.3, C86.30, C86.31, C86.4, C86.40, C86.41, C86.5, C86.50, C86.51, C86.6, C86.60, C86.61, C88, C88.0, C88.00, C88.01, C88.2, C88.20, C88.21, C88.3, C88.30, C88.31, C88.4, C88.40, C88.41, C88.8, C88.80, C88.81, C88.9, C88.90, C88.91, C90, C90.0, C90.00, C90.01, C90.02, C90.1, C90.10, C90.11, C90.12, C90.2, C90.20, C90.21, C90.22, C90.3, C90.30, C90.31, C90.32, C91, C91.0, C91.00, C91.01, C91.02, C91.1, C91.10, C91.11, C91.12, C91.3, C91.30, C91.31, C91.32, C91.4, C91.40, C91.41, C91.42, C91.5, C91.50, C91.51, C91.52, C91.6, C91.60, C91.61, C91.62, C91.9, C91.90, C91.91, C91.92, C91.A, C91.A0, C91.A1, C91.A2, C91.Z, C91.Z0, C91.Z1, C91.Z2, C92, C92.0, C92.00, C92.01, C92.02, C92.1, C92.10, C92.11, C92.12, C92.2, C92.20, C92.21, C92.22, C92.3, C92.30, C92.31, C92.32, C92.4, C92.40, C92.41, C92.42, C92.5, C92.50, C92.51, C92.52, C92.6, C92.60, C92.61, C92.62, C92.9, C92.90, C92.91, C92.92, C92.A, C92.A0, C92.A1, C92.A2, C92.Z, C92.Z0, C92.Z1, C92.Z2, C93, C93.0, C93.00, C93.01, C93.02, C93.1, C93.10, C93.11, C93.12, C93.3, C93.30, C93.31, C93.32, C93.9, C93.90, C93.91, C93.92, C93.Z, C93.Z0, C93.Z1, C93.Z2, C94, C94.0, C94.00, C94.01, C94.02, C94.2, C94.20, C94.21, C94.22, C94.3, C94.30, C94.31, C94.32, C94.4, C94.40, C94.41, C94.42, C94.6, C94.8, C94.80, C94.81, C94.82, C95, C95.0, C95.00, C95.01, C95.02, C95.1, C95.10, C95.11, C95.12, C95.9, C95.90, C95.91, C95.92, C96, C96.0, C96.2, C96.20, C96.21, C96.22, C96.29, C96.4, C96.5, C96.6, C96.9, C96.A, C96.Z, D47.Z1, D59, D59.0, D59.1, D59.10, D59.11, D59.12, D59.13, D59.19, D59.2, D59.3, D59.30, D59.31, D59.32, D59.39, D59.4, D59.5, D59.6, D59.8, D59.9, D60, D60.0, D60.1, D60.8, D60.9, D61, D61.0, D61.01, D61.02, D61.03, D61.09, D61.1, D61.2, D61.3, D61.8, D61.81, D61.810, D61.811, D61.818, D61.82, D61.89, D61.9, D70.0, D71, D72.89, D80, D80.0, D80.1, D80.2, D80.3, D80.4, D80.5, D80.6, D80.7, D80.8, D80.9, D81.0, D81.1, D81.2, D81.4, D81.5, D81.6, D81.7, D81.8, D81.81, D81.810, D81.818, D81.819, D81.82, D81.89, D81.9, D82, D82.0, D82.1, D82.2, D82.3, D82.4, D82.8, D82.9, D83, D83.0, D83.1, D83.2, D83.8, D83.9, D84, D84.0, D84.1, D84.8, D84.81, D84.82, D84.821, D84.822, D84.89, D84.9, D89.0, D89.1, D89.3, D89.4, D89.40, D89.41, D89.42, D89.43, D89.44, D89.49, D89.8, D89.81, D89.810, D89.811, D89.812, D89.813, D89.82, D89.83, D89.831, D89.832, D89.833, D89.834, D89.835, D89.839, D89.84, D89.89, D89.9, L93.0, L93.2, L94, L94.0, L94.1, L94.2, L94.3, L94.4, L94.5, L94.6, L94.8, L94.9, M32, M32.0, M32.1, M32.10, M32.11, M32.12, M32.13, M32.14, M32.15, M32.19, M32.8, M32.9, O98.7, O98.71, O98.711, O98.712, O98.713, O98.719, O98.72, O98.73, T86.0, T86.00, T86.01, T86.02, T86.03, T86.09, T86.1, T86.10, T86.11, T86.12, T86.13, T86.19, T86.2, T86.20, T86.21, T86.22, T86.23, T86.29, T86.290, T86.298, T86.3, T86.30, T86.31, T86.32, T86.33, T86.39, T86.4, T86.40, T86.41, T86.42, T86.43, T86.49, T86.5, T86.81, T86.810, T86.811, T86.812, T86.818, T86.819, T86.85, T86.850, T86.851, T86.852, T86.858, T86.859, Z21, Z48.2, Z48.21, Z48.22, Z48.23, Z48.24, Z48.28, Z48.280, Z48.288, Z48.29, Z48.290, Z48.298, Z51.0, Z51.1, Z51.11, Z51.12, Z94, Z94.0, Z94.1, Z94.2, Z94.3, Z94.4, Z94.5, Z94.6, Z94.7, Z94.8, Z94.81, Z94.82, Z94.83, Z94.84, Z94.89, Z94.9, Z98.85, B21, B21.0, B21.1, B21.2, B21.3, B21.7, B21.8, B21.9, B22, B22.0, B22.1, B22.2, B22.7, B23, B23.1, B23.2, B23.3, B24 |
| Neurological disorders | *Including but not limited to codes for dementia, encephalitis, Huntington’s disease, hereditary ataxia, spinal muscular atrophy, Parkinson’s disease, secondary parkinsonism, dystonia, extrapyramidal and movement disorder, Alzheimer’s disease, multiple sclerosis, facial nerve disorder, carpal tunnel syndrome, mononeuropathy, hereditary and idiopathic neuropathy, inflammatory polyneuropathy, myoneural disorder, primary disorder of muscles, cerebral palsy, hemiplegia and hemiparesis, hydrocephalus, subarachnoid and intracerebral hemorrhage, cerebral infarction, occlusion and stenosis, and cerebrovascular disease.*  **ICD-10-CM:**  F01.50, F01.51, F01.511, F01.518, F03.90, F03.91, F03.911, F03.918, G04, G04.0, G04.00, G04.01, G04.02, G04.1, G04.2, G04.3, G04.30, G04.31, G04.32, G04.39, G04.8, G04.81, G04.82, G04.89, G04.9, G04.90, G04.91, G05, G05.3, G05.4, G10, G11, G11.0, G11.1, G11.10, G11.11, G11.19, G11.2, G11.3, G11.4, G11.5, G11.6, G11.8, G11.9, G12, G12.0, G12.1, G12.2, G12.20, G12.21, G12.22, G12.23, G12.24, G12.25, G12.29, G12.8, G12.9, G13, G13.0, G13.1, G13.2, G13.8, G14, G20, G20.A, G20.A1, G20.A2, G20.B, G20.B1, G20.B2, G20.C, G21, G21.0, G21.1, G21.11, G21.19, G21.2, G21.3, G21.4, G21.8, G21.9, G23, G23.0, G23.1, G23.2, G23.3, G23.8, G23.9, G24, G24.0, G24.01, G24.02, G24.09, G24.1, G24.2, G24.3, G24.4, G24.5, G24.8, G24.9, G25, G25.0, G25.1, G25.2, G25.3, G25.4, G25.5, G25.6, G25.61, G25.69, G25.7, G25.70, G25.71, G25.79, G25.8, G25.81, G25.82, G25.83, G25.89, G25.9, G26, G30, G30.0, G30.1, G30.8, G30.9, G31, G31.0, G31.01, G31.09, G31.1, G31.2, G31.8, G31.80, G31.81, G31.82, G31.83, G31.84, G31.85, G31.86, G31.89, G31.9, G32, G32.0, G32.8, G32.81, G32.89, G35, G36, G36.0, G36.1, G36.8, G36.9, G37, G37.0, G37.1, G37.2, G37.3, G37.4, G37.5, G37.8, G37.81, G37.89, G37.9, G45, G45.0, G45.1, G45.2, G45.3, G45.4, G45.8, G45.9, G46, G46.0, G46.1, G46.2, G46.3, G46.4, G46.5, G46.6, G46.7, G46.8, G50, G50.0, G50.1, G50.8, G50.9, G51, G51.0, G51.1, G51.2, G51.3, G51.31, G51.32, G51.33, G51.39, G51.4, G51.8, G51.9, G52, G52.0, G52.1, G52.2, G52.3, G52.7, G52.8, G52.9, G53, G54, G54.0, G54.1, G54.2, G54.3, G54.4, G54.5, G54.6, G54.7, G54.8, G54.9, G55, G56, G56.0, G56.00, G56.01, G56.02, G56.03, G56.1, G56.10, G56.11, G56.12, G56.13, G56.2, G56.20, G56.21, G56.22, G56.23, G56.3, G56.30, G56.31, G56.32, G56.33, G56.4, G56.40, G56.41, G56.42, G56.43, G56.8, G56.80, G56.81, G56.82, G56.83, G56.9, G56.90, G56.91, G56.92, G56.93, G57, G57.0, G57.00, G57.01, G57.02, G57.03, G57.1, G57.10, G57.11, G57.12, G57.13, G57.2, G57.20, G57.21, G57.22, G57.23, G57.3, G57.30, G57.31, G57.32, G57.33, G57.4, G57.40, G57.41, G57.42, G57.43, G57.5, G57.50, G57.51, G57.52, G57.53, G57.6, G57.60, G57.61, G57.62, G57.63, G57.7, G57.70, G57.71, G57.72, G57.73, G57.8, G57.80, G57.81, G57.82, G57.83, G57.9, G57.90, G57.91, G57.92, G57.93, G58, G58.0, G58.7, G58.8, G58.9, G59, G60, G60.0, G60.1, G60.2, G60.3, G60.8, G60.9, G61, G61.0, G61.1, G61.8, G61.81, G61.82, G61.89, G61.9, G62, G62.0, G62.1, G62.2, G62.8, G62.81, G62.82, G62.89, G62.9, G63, G64, G65, G65.0, G65.1, G65.2, G70, G70.0, G70.00, G70.01, G70.1, G70.2, G70.8, G70.80, G70.81, G70.89, G70.9, G71, G71.0, G71.00, G71.01, G71.02, G71.03, G71.031, G71.032, G71.033, G71.034, G71.0340, G71.0341, G71.0342, G71.0349, G71.035, G71.038, G71.039, G71.09, G71.1, G71.11, G71.12, G71.13, G71.14, G71.19, G71.2, G71.20, G71.21, G71.22, G71.220, G71.228, G71.29, G71.3, G71.8, G71.9, G72, G72.0, G72.1, G72.2, G72.3, G72.4, G72.41, G72.49, G72.8, G72.81, G72.89, G72.9, G73, G73.1, G73.3, G73.7, G80, G80.0, G80.1, G80.2, G80.3, G80.4, G80.8, G80.9, G81, G81.0, G81.00, G81.01, G81.02, G81.03, G81.04, G81.1, G81.10, G81.11, G81.12, G81.13, G81.14, G81.9, G81.90, G81.91, G81.92, G81.93, G81.94, G82, G82.2, G82.20, G82.21, G82.22, G82.5, G82.50, G82.51, G82.52, G82.53, G82.54, G83, G83.0, G83.1, G83.10, G83.11, G83.12, G83.13, G83.14, G83.2, G83.20, G83.21, G83.22, G83.23, G83.24, G83.3, G83.30, G83.31, G83.32, G83.33, G83.34, G83.4, G83.5, G83.8, G83.81, G83.82, G83.83, G83.84, G83.89, G83.9, G91, G91.0, G91.1, G91.2, G91.3, G91.4, G91.8, G91.9, G93.1, G93.4, G93.40, G93.41, G93.42, G93.43, G93.44, G93.45, G93.49, G95, G95.0, G95.1, G95.11, G95.19, G95.2, G95.20, G95.29, G95.8, G95.81, G95.89, G95.9, I60, I60.0, I60.00, I60.01, I60.02, I60.1, I60.10, I60.11, I60.12, I60.2, I60.20, I60.21, I60.22, I60.3, I60.30, I60.31, I60.32, I60.4, I60.5, I60.50, I60.51, I60.52, I60.6, I60.7, I60.8, I60.9, I61, I61.0, I61.1, I61.2, I61.3, I61.4, I61.5, I61.6, I61.8, I61.9, I62, I62.0, I62.00, I62.01, I62.02, I62.03, I62.1, I62.9, I63, I63.0, I63.00, I63.01, I63.011, I63.012, I63.013, I63.019, I63.02, I63.03, I63.031, I63.032, I63.033, I63.039, I63.09, I63.1, I63.10, I63.11, I63.111, I63.112, I63.113, I63.119, I63.12, I63.13, I63.131, I63.132, I63.133, I63.139, I63.19, I63.2, I63.20, I63.21, I63.211, I63.212, I63.213, I63.219, I63.22, I63.23, I63.231, I63.232, I63.233, I63.239, I63.29, I63.3, I63.30, I63.31, I63.311, I63.312, I63.313, I63.319, I63.32, I63.321, I63.322, I63.323, I63.329, I63.33, I63.331, I63.332, I63.333, I63.339, I63.34, I63.341, I63.342, I63.343, I63.349, I63.39, I63.4, I63.40, I63.41, I63.411, I63.412, I63.413, I63.419, I63.42, I63.421, I63.422, I63.423, I63.429, I63.43, I63.431, I63.432, I63.433, I63.439, I63.44, I63.441, I63.442, I63.443, I63.449, I63.49, I63.5, I63.50, I63.51, I63.511, I63.512, I63.513, I63.519, I63.52, I63.521, I63.522, I63.523, I63.529, I63.53, I63.531, I63.532, I63.533, I63.539, I63.54, I63.541, I63.542, I63.543, I63.549, I63.59, I63.6, I63.8, I63.81, I63.89, I63.9, I65, I65.0, I65.01, I65.02, I65.03, I65.09, I65.1, I65.2, I65.21, I65.22, I65.23, I65.29, I65.8, I65.9, I66, I66.0, I66.01, I66.02, I66.03, I66.09, I66.1, I66.11, I66.12, I66.13, I66.19, I66.2, I66.21, I66.22, I66.23, I66.29, I66.3, I66.8, I66.9, I67, I67.0, I67.1, I67.2, I67.3, I67.4, I67.5, I67.6, I67.7, I67.8, I67.81, I67.82, I67.83, I67.84, I67.841, I67.848, I67.85, I67.850, I67.858, I67.89, I67.9, I68, I68.0, I68.2, I68.8, I69, I69.0, I69.00, I69.01, I69.010, I69.011, I69.012, I69.013, I69.014, I69.015, I69.018, I69.019, I69.02, I69.020, I69.021, I69.022, I69.023, I69.028, I69.03, I69.031, I69.032, I69.033, I69.034, I69.039, I69.04, I69.041, I69.042, I69.043, I69.044, I69.049, I69.05, I69.051, I69.052, I69.053, I69.054, I69.059, I69.06, I69.061, I69.062, I69.063, I69.064, I69.065, I69.069, I69.09, I69.090, I69.091, I69.092, I69.093, I69.098, I69.1, I69.10, I69.11, I69.110, I69.111, I69.112, I69.113, I69.114, I69.115, I69.118, I69.119, I69.12, I69.120, I69.121, I69.122, I69.123, I69.128, I69.13, I69.131, I69.132, I69.133, I69.134, I69.139, I69.14, I69.141, I69.142, I69.143, I69.144, I69.149, I69.15, I69.151, I69.152, I69.153, I69.154, I69.159, I69.16, I69.161, I69.162, I69.163, I69.164, I69.165, I69.169, I69.19, I69.190, I69.191, I69.192, I69.193, I69.198, I69.2, I69.20, I69.21, I69.210, I69.211, I69.212, I69.213, I69.214, I69.215, I69.218, I69.219, I69.22, I69.220, I69.221, I69.222, I69.223, I69.228, I69.23, I69.231, I69.232, I69.233, I69.234, I69.239, I69.24, I69.241, I69.242, I69.243, I69.244, I69.249, I69.25, I69.251, I69.252, I69.253, I69.254, I69.259, I69.26, I69.261, I69.262, I69.263, I69.264, I69.265, I69.269, I69.29, I69.290, I69.291, I69.292, I69.293, I69.298, I69.3, I69.30, I69.31, I69.310, I69.311, I69.312, I69.313, I69.314, I69.315, I69.318, I69.319, I69.32, I69.320, I69.321, I69.322, I69.323, I69.328, I69.33, I69.331, I69.332, I69.333, I69.334, I69.339, I69.34, I69.341, I69.342, I69.343, I69.344, I69.349, I69.35, I69.351, I69.352, I69.353, I69.354, I69.359, I69.36, I69.361, I69.362, I69.363, I69.364, I69.365, I69.369, I69.39, I69.390, I69.391, I69.392, I69.393, I69.398, I69.8, I69.80, I69.81, I69.810, I69.811, I69.812, I69.813, I69.814, I69.815, I69.818, I69.819, I69.82, I69.820, I69.821, I69.822, I69.823, I69.828, I69.83, I69.831, I69.832, I69.833, I69.834, I69.839, I69.84, I69.841, I69.842, I69.843, I69.844, I69.849, I69.85, I69.851, I69.852, I69.853, I69.854, I69.859, I69.86, I69.861, I69.862, I69.863, I69.864, I69.865, I69.869, I69.89, I69.890, I69.891, I69.892, I69.893, I69.898, I69.9, I69.90, I69.91, I69.910, I69.911, I69.912, I69.913, I69.914, I69.915, I69.918, I69.919, I69.92, I69.920, I69.921, I69.922, I69.923, I69.928, I69.93, I69.931, I69.932, I69.933, I69.934, I69.939, I69.94, I69.941, I69.942, I69.943, I69.944, I69.949, I69.95, I69.951, I69.952, I69.953, I69.954, I69.959, I69.96, I69.961, I69.962, I69.963, I69.964, I69.965, I69.969, I69.99, I69.990, I69.991, I69.992, I69.993, I69.998  *G35 additionally requires the non-occurrence of immunotherapy/chemotherapy in the baseline period.* |
| Obesity | *Including codes for obesity and body mass index of 30 or higher.*  **ICD-10-CM:**  E66, E66.0, E66.01, E66.09, E66.1, E66.2, E66.8, E66.81, E66.811, E66.812, E66.813, E66.89, E66.9, Z68.3, Z68.30, Z68.31, Z68.32, Z68.33, Z68.34, Z68.35, Z68.36, Z68.37, Z68.38, Z68.39, Z68.4, Z68.41, Z68.42, Z68.43, Z68.44, Z68.45 |
| Immunotherapies/Chemotherapies | **Generic drug names:**  ABATACEPT, ABATACEPT/MALTOSE, ABEMACICLIB, ACALABRUTINIB, ACALABRUTINIB MALEATE, ADALIMUMAB, ADALIMUMAB-AACF, ADALIMUMAB-AATY, ADALIMUMAB-ADAZ, ADALIMUMAB-ADBM, ADALIMUMAB-AFZB, ADALIMUMAB-AQVH, ADALIMUMAB-ATTO, ADALIMUMAB-BWWD, ADALIMUMAB-FKJP, ADALIMUMAB-RYVK, ADO-TRASTUZUMAB EMTANSINE, AFATINIB DIMALEATE, ALECTINIB HCL, ALEFACEPT, ALEMTUZUMAB, ALPELISIB, ALTRETAMINE, ANAKINRA, APREMILAST, ARSENIC TRIOXIDE, ASCIMINIB HYDROCHLORIDE, ASPARAGINASE, ASPARAGINASE (ERWINIA CHRYSANTHEMI), ASPARAGINASE ERWINIA CHRYSANTHEMI (RECOMBINANT)-RYWN, AVAPRITINIB, AVUTOMETINIB POTASSIUM, AVUTOMETINIB POTASSIUM/DEFACTINIB HYDROCHLORIDE, AXITINIB, AZACITIDINE, AZATHIOPRINE, AZATHIOPRINE SODIUM, BARICITINIB, BASILIXIMAB, BELATACEPT, BELIMUMAB, BELINOSTAT, BELUMOSUDIL MESYLATE, BELZUTIFAN, BENDAMUSTINE HCL, BEVACIZUMAB, BEXAROTENE, BINIMETINIB, BLEOMYCIN SULFATE, BORTEZOMIB, BOSUTINIB, BRIGATINIB, BRODALUMAB, BUSULFAN, CABAZITAXEL, CABOZANTINIB S-MALATE, CALASPARGASE PEGOL-MKNL, CANAKINUMAB/PF, CAPECITABINE, CAPIVASERTIB, CAPMATINIB HYDROCHLORIDE, CARBOPLATIN, CARFILZOMIB, CARMUSTINE, CARMUSTINE IN POLIFEPROSAN 20, CERITINIB, CERTOLIZUMAB PEGOL, CETUXIMAB, CHLORAMBUCIL, CISPLATIN, CLADRIBINE, CLOFARABINE, COBIMETINIB FUMARATE, COPANLISIB DI-HCL, CRIZOTINIB, CYCLOPHOSPHAMIDE, CYCLOSPORINE, CYCLOSPORINE, MODIFIED, CYCLOSPORINE/CHONDROITIN SULFATE A SODIUM, CYSTEAMINE BITARTRATE, CYTARABINE, CYTARABINE LIPOSOME/PF, CYTARABINE/PF, DABRAFENIB MESYLATE, DACARBAZINE, DACLIZUMAB, DACOMITINIB, DACTINOMYCIN, DASATINIB, DAUNORUBICIN CITRATE LIPOSOMAL, DAUNORUBICIN HCL, DAUNORUBICIN/CYTARABINE LIPOSOMAL, DECITABINE, DECITABINE/CEDAZURIDINE, DENILEUKIN DIFTITOX, DEURUXOLITINIB PHOSPHATE, DEXAMETHASONE, DEXAMETHASONE ACETATE, DEXAMETHASONE ACETATE AND SODIUM PHOSPHATE IN STERILE WATER, DEXAMETHASONE ACETATE IN SODIUM CHLORIDE, ISO-OSMOTIC, DEXAMETHASONE ACETATE, MICRONIZED, DEXAMETHASONE ISONICOTINATE, DEXAMETHASONE PHOSPHATE, DEXAMETHASONE SODIUM PHOSPHATE, DEXAMETHASONE SODIUM PHOSPHATE IN 0.9 % SODIUM CHLORIDE, DEXAMETHASONE SODIUM PHOSPHATE/LIDOCAINE HCL, DEXAMETHASONE SODIUM PHOSPHATE/PF, DEXAMETHASONE, MICRONIZED, DEXAMETHASONE/PF, DILUENT FOR CARMUSTINE (ETHANOL), DILUENT FOR DECITABINE (POTASS PH MONOBASIC,SODIUM HYDROX), DILUENT FOR MELPHALAN (SODIUM CITRATE), DILUENT FOR TEMSIROLIMUS (ETHANOL), DIMETHYL FUMARATE, DIROXIMEL FUMARATE, DOCETAXEL, DOXORUBICIN HCL, DOXORUBICIN HCL PEGYLATED LIPOSOMAL, DUVELISIB, ECULIZUMAB, EFALIZUMAB, EMAPALUMAB-LZSG, ENASIDENIB MESYLATE, ENCORAFENIB, ENTRECTINIB, ERDAFITINIB, ERIBULIN MESYLATE, ERLOTINIB HCL, ESTRAMUSTINE PHOSPHATE SODIUM, ETANERCEPT, ETOPOSIDE, ETOPOSIDE PHOSPHATE, EVEROLIMUS, FAM-TRASTUZUMAB DERUXTECAN-NXKI, FEDRATINIB DIHYDROCHLORIDE, FINGOLIMOD HCL, FLOXURIDINE, FLUDARABINE PHOSPHATE, FLUOROURACIL, FLUOROURACIL/ADHESIVE BANDAGE, FLUOROURACIL/CALCIPOTRIENE, FRUQUINTINIB, FUTIBATINIB, GEFITINIB, GEMCITABINE HCL, GEMCITABINE HCL IN 0.9 % SODIUM CHLORIDE, GEMTUZUMAB OZOGAMICIN, GILTERITINIB FUMARATE, GLASDEGIB MALEATE, GOLIMUMAB, GUSELKUMAB, HYDROCORTISONE, HYDROCORTISONE ACETATE, HYDROCORTISONE BUTYRATE, HYDROCORTISONE CYPIONATE, HYDROCORTISONE HEMISUCCINATE, HYDROCORTISONE PROBUTATE, HYDROCORTISONE SOD PHOSPHATE, HYDROCORTISONE SODIUM SUCCINATE, HYDROCORTISONE SODIUM SUCCINATE/PF, HYDROCORTISONE VALERATE, HYDROXYUREA, IBRUTINIB, IDARUBICIN HCL, IDELALISIB, IFOSFAMIDE, IFOSFAMIDE/MESNA, IMATINIB MESYLATE, IMETELSTAT SODIUM, INAVOLISIB, INEBILIZUMAB-CDON, INFIGRATINIB PHOSPHATE, INFLIXIMAB, INFLIXIMAB-ABDA, INFLIXIMAB-AXXQ, INFLIXIMAB-DYYB, IOBENGUANE IODINE-131, IRINOTECAN HCL, IRINOTECAN LIPOSOMAL, IVOSIDENIB, IXABEPILONE, IXAZOMIB CITRATE, IXEKIZUMAB, KIT FOR PREP INDIUM-111/IBRITUMOMAB TIUXETAN/ALBUMIN HUMAN, KIT FOR PREP YTTRIUM-90/IBRITUMOMAB TIUXETAN/ALBUMIN HUMAN, LAPATINIB DITOSYLATE, LAROTRECTINIB SULFATE, LAZERTINIB MESYLATE, LEFLUNOMIDE, LENALIDOMIDE, LENVATINIB MESYLATE, LETROZOLE, LOMUSTINE, LORLATINIB, LURBINECTEDIN, LUTETIUM LU 177 DOTATATE, LYMPHOCYTE IG,ANTITHYMOCYT,EQU/THIMEROSAL, LYMPHOCYTE IMMUNE GLOBULIN,ANTITHYMOCYTE (EQUINE), MECHLORETHAMINE HCL, MEGESTROL ACETATE, MEGESTROL ACETATE,MICRONIZED, MELPHALAN, MELPHALAN FLUFENAMIDE HYDROCHLORIDE, MELPHALAN HCL, MELPHALAN HCL/BETADEX SULFOBUTYL ETHER SODIUM, MERCAPTOPURINE, METHOTREXATE, METHOTREXATE SODIUM, METHOTREXATE SODIUM/PF, METHOTREXATE/PF, METHOXSALEN, METHYLPREDNISOLONE, METHYLPREDNISOLONE ACETATE, METHYLPREDNISOLONE ACETATE IN SODIUM CHLORIDE,ISO-OSMOTIC/PF, METHYLPREDNISOLONE ACETATE IN STERILE WATER FOR INJECTION, METHYLPREDNISOLONE ACETATE, MICRONIZED, METHYLPREDNISOLONE SODIUM SUCCINATE, METHYLPREDNISOLONE SODIUM SUCCINATE/PF, METHYLPREDNISOLONE, MICRONIZED, MITOMYCIN, MITOTANE, MITOXANTRONE HCL, MOBOCERTINIB SUCCINATE, MOMELOTINIB DIHYDROCHLORIDE, MUROMONAB-CD3, MYCOPHENOLATE MOFETIL, MYCOPHENOLATE MOFETIL HCL, MYCOPHENOLATE SODIUM, NATALIZUMAB, NELARABINE, NERATINIB MALEATE, NILOTINIB HCL, NILOTINIB TARTRATE, NINTEDANIB ESYLATE, NIRAPARIB TOSYLATE, NIRAPARIB TOSYLATE/ABIRATERONE ACETATE, OCRELIZUMAB, OFATUMUMAB, OLAPARIB, OLUTASIDENIB, OMACETAXINE MEPESUCCINATE, OSIMERTINIB MESYLATE, OXALIPLATIN, OZANIMOD HYDROCHLORIDE, PACLITAXEL, PACLITAXEL PROTEIN-BOUND, PACRITINIB CITRATE, PALBOCICLIB, PANITUMUMAB, PANOBINOSTAT LACTATE, PAZOPANIB HCL, PEGASPARGASE, PEMETREXED, PEMETREXED DIPOTASSIUM, PEMETREXED DISODIUM, PEMIGATINIB, PENTOSTATIN, PERTUZUMAB-TRASTUZUMAB-HYALURONIDASE-ZZXF, PEXIDARTINIB HYDROCHLORIDE, PIRFENIDONE, PIRTOBRUTINIB, PLICAMYCIN, POMALIDOMIDE, PONATINIB HCL, PORFIMER SODIUM, PRALATREXATE, PRALSETINIB, PREDNISOLONE, PREDNISOLONE ACETATE, PREDNISOLONE ACETATE, MICRONIZED, PREDNISOLONE ACETATE/PF, PREDNISOLONE SODIUM PHOSPHATE, PREDNISOLONE, MICRONIZED, PREDNISONE, PREDNISONE MICRONIZED, PROCARBAZINE HCL, QUIZARTINIB DIHYDROCHLORIDE, RADIUM-223 DICHLORIDE, RAVULIZUMAB-CWVZ, REGORAFENIB, REPOTRECTINIB, REVUMENIB CITRATE, RIBOCICLIB SUCCINATE, RIBOCICLIB SUCCINATE/LETROZOLE, RILONACEPT, RIPRETINIB, RISANKIZUMAB-RZAA, RITUXIMAB, RITUXIMAB-ABBS, RITUXIMAB-ARRX, RITUXIMAB-PVVR, RITUXIMAB/HYALURONIDASE, HUMAN RECOMBINANT, ROMIDEPSIN, RUCAPARIB CAMSYLATE, RUXOLITINIB PHOSPHATE, SARILUMAB, SATRALIZUMAB-MWGE, SECUKINUMAB, SELINEXOR, SELPERCATINIB, SELUMETINIB SULFATE, SILTUXIMAB, SIPONIMOD, SIROLIMUS, SODIUM IODIDE-131, SONIDEGIB PHOSPHATE, SORAFENIB TOSYLATE, SOTORASIB, STREPTOZOCIN, SUNITINIB MALATE, TACROLIMUS, TACROLIMUS ANHYDROUS, TACROLIMUS IN VEHICLE BASE NO.238, TACROLIMUS, MICRONIZED, TACROLIMUS/HYALURONATE SODIUM/NIACINAMIDE, TACROLIMUS/NIACINAMIDE, TALAZOPARIB TOSYLATE, TALETRECTINIB ADIPATE, TAZEMETOSTAT HYDROBROMIDE, TEMOZOLOMIDE, TEMSIROLIMUS, TENIPOSIDE, TEPOTINIB HCL, TEPROTUMUMAB-TRBW, TERIFLUNOMIDE, THALIDOMIDE, THIOGUANINE, THIOTEPA, TILDRAKIZUMAB-ASMN, TIVOZANIB HCL, TOCILIZUMAB, TOFACITINIB CITRATE, TOPOTECAN HCL, TOVORAFENIB, TRABECTEDIN, TRAMETINIB, TRASTUZUMAB, TRASTUZUMAB-ANNS, TRASTUZUMAB-DKST, TRASTUZUMAB-DTTB, TRASTUZUMAB-HYALURONIDASE-OYSK, TRASTUZUMAB-PKRB, TRASTUZUMAB-QYYP, TRASTUZUMAB-STRF, TREOSULFAN, TRETINOIN, TRIFLURIDINE, TRIFLURIDINE/TIPIRACIL HCL, TRILACICLIB DIHYDROCHLORIDE, TUCATINIB, UMBRALISIB TOSYLATE, UPADACITINIB, USTEKINUMAB, VALRUBICIN, VANDETANIB, VEDOLIZUMAB, VEMURAFENIB, VENETOCLAX, VIMSELTINIB, VINBLASTINE SULFATE, VINCRISTINE SULFATE, VINCRISTINE SULFATE LIPOSOMAL, VINORELBINE TARTRATE, VISMODEGIB, VORASIDENIB CITRATE, VORINOSTAT, ZANUBRUTINIB, ZIV-AFLIBERCEPT, (DAUNORUBICIN AND CYTARABINE) LIPOSOME, AFATINIB, ALECTINIB HYDROCHLORIDE, ANTI-LYMPHOCYTE GLOBULIN, ANTITHYMOCYTE GLOBULIN EQUINE, ANTITHYMOCYTE GLOBULIN RABBIT, ASCIMINIB, ASPARAGINASE (E. COLI), AVUTOMETINIB POTASSIUM AND DEFACTINIB HYDROCHLORIDE, AZACITIDINE FOR, BELUMOSUDIL, BENDAMUSTINE HYDROCHLORIDE, BLEOMYCIN, BORTEXOMIB, BOSUTINIB MONOHYDRATE, BOSUTINIB, CABOZANTINIB, CALASPARGASE PEGOL, CANAKINUMAB, CAPMATINIB, CARBOPLATIN INJECTION, CARMUSTINE IMPLANT/ POLIFEPROSAN 20, POLIFEPROSAN 20 WITH CARMUSTINE IMPLANT, CEDAZURIDINE AND DECITABINE, COBIMETINIB, COPANLISIB, CYCLOPHOSPHAMIDE FOR INJECTION, CYCLOPHOSPHAMIDE INJECTION, SOLUTION, DABRAFENIB, DAUNORUBICIN, DAUNORUBICIN HYDROCHLORIDE, DEXAMETHASONE ISONICOTINATE, MICRONIZED, DEXAMETHASONE SODIUM PHOSPHATE/LIDOCAINE HYDROCHLORIDE, DOCETAXEL ANHYDROUS, DOXORUBICIN, DOXORUBICIN HYDROCHLORIDE, DOXORUBICIN HYDROCHLORIDE LIPOSOME, ENSARTINIB, EPIRUBICIN HYDROCHLORIDE, ERLOTINIB, ERLOTINIB HYDROCHLORIDE, FEDRATINIB HYDROCHLORIDE, FLUDARABINE, FLUOROURACIL CREAM, 0.5%, GEMCITABINE, GEMCITABINE HYDROCHLORIDE, GILTERITINIB, GLASDEGIB, HYDROCORTISONE ACETATE, MICRONIZED, I 131 MINI, IBRITUMOMAB TIUXETAN, IBRITUMOMAB TIUXETAN/SODIUM ACETATE, IDARUBICIN HYDROCHLORIDE, IFOSFAMIDE AND MESNA, IMATINIB, IMATINIB ORAL, INFIGRATINIB, IOBENGUANE I-131, IRINOTECAN, IRINOTECAN HYDROCHLOIDE, IRINOTECAN HYDROCHLORIDE, IXAZOMIB, LAPATINIB, LAROTRECTINIB, LAZERTINIB, LENVATINIB, LETROZOLE AND RIBOCICLIB, MASOPROCOL, MECHLORETHAMINE HYDROCHLORIDE, MEGESTROL ACETATE, MICRONIZED, MELPHALAN FLUFENAMIDE, MELPHALAN HYDROCHLORIDE, MELPHALAN HYDROCHLORIDE INJECTION, POWDER, LYOPHILIZED, FOR SOLUTION, MELPHALAN USP, 2 MG, METHOTREXATE, METHOTREXATE SODIUM, MITOXANTRONE, MITOXANTRONE HYDROCHLORIDE, MOBOCERTINIB, MOMELOTINIB, MYCOPHENOLATE, NELARABINE INJECTION, NERATINIB, NILOTINIB, NILOTINIB HYDROCHLORIDE, NINTEDANIB, NIRAPARIB, NIRAPARIB TOSYLATE MONOHYDRATE AND ABIRATERONE ACETATE, OSIMERTINIB, P32 SODIUM PHOSPHATE, PACLITAXEL PROTEIN-BOUND PARTICLES, PACRITINIB, PANOBINOSTAT, PAZOPANIB, PAZOPANIB HYDROCHLORIDE, PEXIDARTINIB, PIPOBROMAN, PONATINIB HYDROCHLORIDE, PROCARBAZINE HYDROCHLORIDE, PURIXAN, QUIZARTINIB, RADIUM RA 223 DICHLORIDE, REVUMENIB, RIBOCICLIB, RUCAPARIB, RUXOLITINIB, RYDAPT, SELUMETINIB, SODIUM IODIDE I 131, SODIUM IODIDE I-131, SODIUM IODIDE, I-131, SONIDEGIB, SORAFENIB, TALAZOPARIB, TALETRECTINIB, TAZEMETOSTAT, TEMSIROLIMUS INJECTION, TEPOTINIB HYDROCHLORIDE, TIVOZANIB, TOPOTECAN, TOPOTECAN HYDROCHLORIDE, TRIFLURIDINE AND TIPIRACIL, TRILACICLIB, UMBRALISIB, VALRUBICIN INTRAVESICAL SOLUTION, VINORELBINE, VORASIDENIB |
| Antibiotic medications | **Generic drug names:**  AZITHROMYCIN, ERYTHROMYCIN, RIFAXIMIN, CIPROFLOXACIN, LEVOFLOXACIN, TRIMETHOPRIM-SULFAMETHOXAZOLE, METRONIDAZOLE, NITAZOXANIDE, AMOXICILLIN, CEFIXIME, CEFTRIAXONE, DOXYCYCLINE, CEFDINIR, AMOXICILLIN-CLAVULANATE, FIDAXOMICIN, VANCOMYCIN, PIPERACILLIN-TAZOBACTAM, CEFEPIME, MEROPENEM  **HCPCS:**  G9315, J0456, J0692, J0696, J0701, J0703, J0744, J1271, J1364, J1836, J1956, J2183, J2184, J2185, J2186, J3370, J3371, J3372, J3373, J3374, J3375, J3376, Q0144 |
| Antidiarrheal medications | **Generic drug names:**  LOPERAMIDE, DIPHENOXYLATE AND ATROPINE, BISMUTH SUBSALICYLATE, CHOLESTYRAMINE, OCTREOTIDE  **HCPCS:**  J2353, J2354 |
| Antiemetic medications | **Generic drug names:**  METOCLOPRAMIDE, ONDANSETRON, PROCHLORPERAZINE, PROMETHAZINE, GRANISETRON, SCOPOLAMINE, APREPITANT  **HCPCS:**  C9145, J0185, J0780, J1626, J1627, J2405, J2550, J2765, J8498, J8501, Q0162, Q0164, Q0166, Q0169, S0091, S0119, S0183 |
| Charlson-Quan Comorbidity Index | *ICD-10-CM codes for the Charlson-Quan Comorbidity Index were defined according to the methodology outlined by Quan et al. (2005). The specifics are not reproduced here and should be referenced directly from the original source.*  *Citation: Quan H, Sundararajan V, Halfon P, Fong A, Burnand B, Luthi JC, Saunders LD, Beck CA, Feasby TE, Ghali WA. Coding algorithms for defining comorbidities in ICD-9-CM and ICD-10 administrative data. Med Care. 2005 Nov;43(11):1130-9. doi: 10.1097/01.mlr.0000182534.19832.83.* |

Abbreviations: CPT, Current Procedural Terminology; HCPCS, Healthcare Common Procedure Coding System; ICD-10-CM, International Classification of Diseases, 10th Revision, Clinical Modification


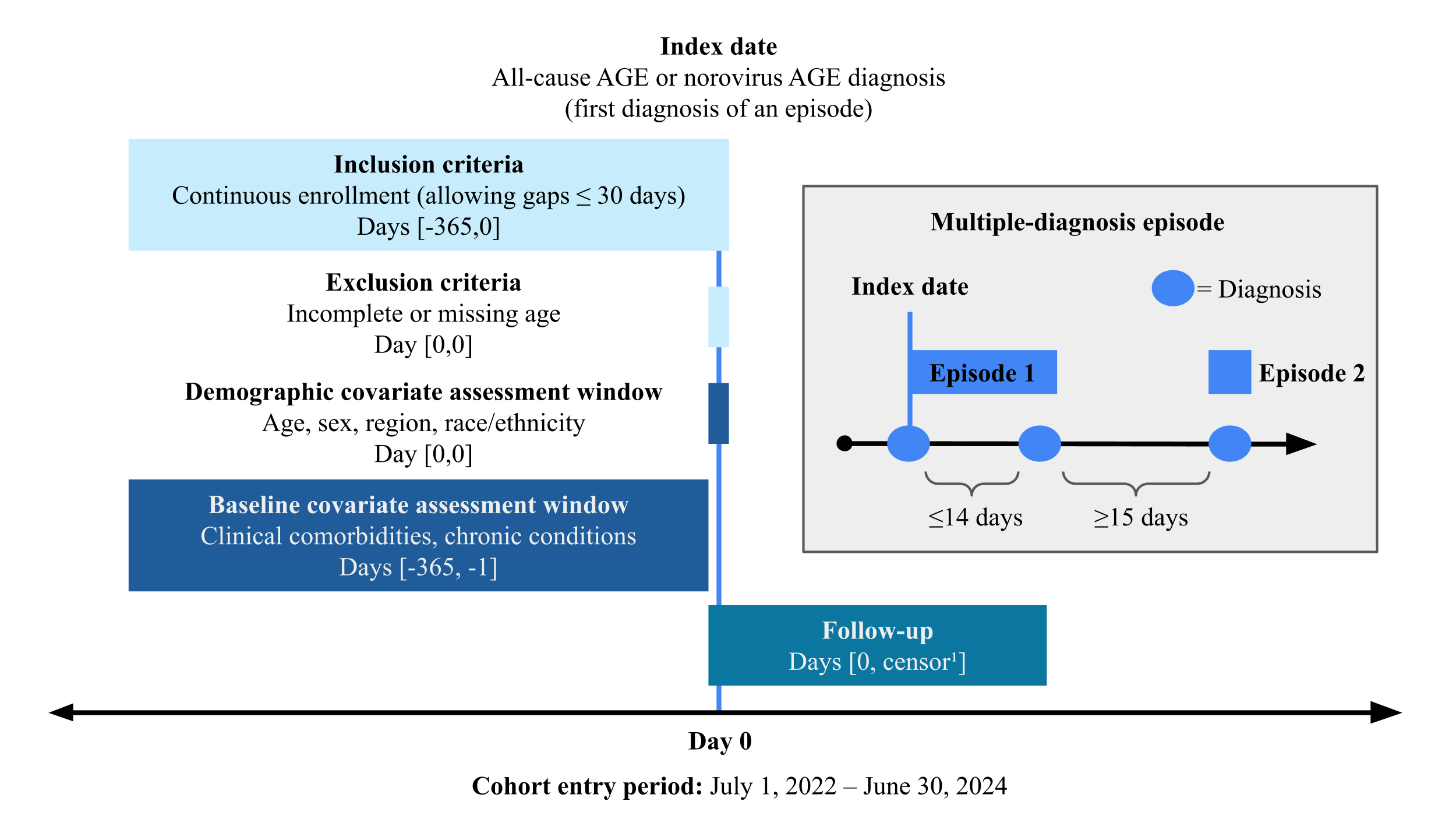


**Supplementary Figure 1:** The **s**tudy design diagram illustrates the index date, eligibility and covariate assessment windows, and follow-up period for the identification of acute hospitalization or other healthcare resource utilization. The embedded diagram on the right indicates the algorithm for an episode of all-cause AGE or norovirus AGE: multiple claims with AGE diagnosis codes within 14 days of one another were considered part of a single AGE episode. *Abbreviation: AGE, acute gastroenteritis.* ^1^Follow-up is censored on the earliest of the following: day 3 of follow-up, disenrollment, death, or end of study period (June 30, 2024).


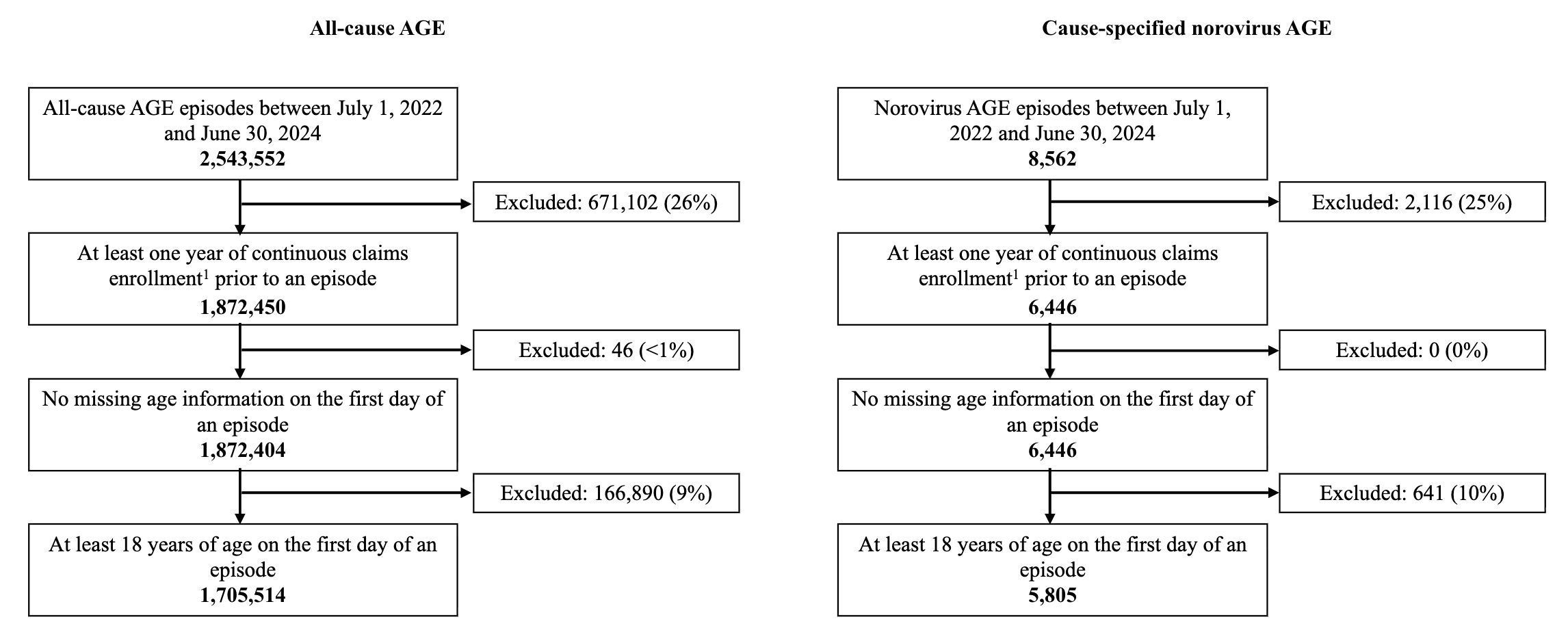


**Supplementary Figure 2:** The study population attrition diagrams describe the number and percentage of episodes excluded by each eligibility criterion, resulting in 1,705,514 all-cause AGE episodes^2^ and 5,805 norovirus AGE episodes^2^ for final analysis. All episodes were identified from Optum’s de-identified Clinformatics® Data Mart Database (Optum® CDM). *Abbreviation: AGE, acute gastroenteritis.* ^1^Continuous claims enrollment is defined as enrollment from 365 days prior to index date (i.e., the first day of an episode of all-cause AGE or norovirus AGE) through the index date, with enrollment gaps of up to 30 days allowed. ^2^Multiple claims with all-cause AGE or norovirus AGE diagnosis codes within 14 days of one another were considered part of a single all-cause AGE or norovirus AGE episode, respectively.

**Supplementary Table 2.** Acute hospitalization and other healthcare resource utilization after all-cause acute gastroenteritis and norovirus acute gastroenteritis episodes, stratified by age

|  | **All-cause AGE** | | | | | | **Norovirus AGE** | | | | | |
| --- | --- | --- | --- | --- | --- | --- | --- | --- | --- | --- | --- | --- |
|  | **18–64 years** | | | **≥65 years** | | | **18–64 years** | | | **≥65 years** | | |
|  | **Overall** | **With underlying medical condition** | **Without underlying medical condition** | **Overall** | **With underlying medical condition** | **Without underlying medical condition** | **Overall** | **With underlying medical condition** | **Without underlying medical condition** | **Overall** | **With underlying medical condition** | **Without underlying medical condition** |
| **Total number of unique adult patients, n** | 449,689 | 274,688 | 193,435 | 664,039 | 611,361 | 66,195 | 1,752 | 1,195 | 561 | 3,615 | 3,471 | 147 |
| **Total number of eligible episodes^1^** | 641,632 | 418,958 | 222,674 | 1,063,882 | 986,092 | 77,790 | 1,911 | 1,347 | 564 | 3,894 | 3,746 | 148 |
| **Healthcare resource utilization** | | | | | | | | | | | | |
| **Acute hospitalization within 3 days on or after index date** | | | | | | | | | | | | |
| **Acute hospitalization; n (%)** | 39,748 (6.19%) | 35,239 (8.41%) | 4,509 (2.02%) | 152,682 (14.35%) | 146,728 (14.88%) | 5,954 (7.65%) | 600 (31.40%) | 521 (38.68%) | 79 (14.01%) | 1,989 (51.08%) | 1,936 (51.68%) | 53 (35.81%) |
| **Length of stay^2^** | | | | | | | | | | | | |
| Mean (SD) | 7.16 (8.97) | 7.26 (9.08) | 6.43 (7.97) | 7.30 (9.09) | 7.27 (9.06) | 7.89 (9.74) | 7.09 (7.68) | 7.44 (7.93) | 4.76 (5.18) | 8.44 (15.94) | 8.51 (16.11) | 5.91 (7.18) |
| Median [IQR] | 5.00 [3.00–8.00] | 5.00 [3.00–8.00] | 4.00 [3.00–7.00] | 5.00 [3.00–8.00] | 5.00 [3.00–8.00] | 5.00 [4.00–9.00] | 5.00 [3.00–7.25] | 5.00 [4.00–8.00] | 3.00 [3.00–4.00] | 5.00 [4.00–9.00] | 5.00 [4.00–9.00] | 4.00 [3.00–5.00] |
| **ICU admission; n (%) out of patients with acute hospitalization^2^** | 14,544 (36.59%) | 13,283 (37.69%) | 1,261 (27.97%) | 59,337 (38.86%) | 57,092 (38.91%) | 2,245 (37.71%) | 203 (33.83%) | 186 (35.70%) | 17 (21.52%) | 682 (34.29%) | 666 (34.40%) | 16 (30.19%) |
| **Use of antiemetics, antidiarrheal agents, and antibiotics within 3 days on or after index date; n (%)** | | | | | | | | | | | | |
| Overall use | 190,930 (29.76%) | 117,187 (27.97%) | 73,743 (33.12%) | 207,167 (19.47%) | 190,273 (19.30%) | 16,894 (21.72%) | 764 (39.98%) | 473 (35.12%) | 291 (51.60%) | 996 (25.58%) | 935 (24.96%) | 61 (41.22%) |
| Use of antiemetics | 141,259 (22.02%) | 85,004 (20.29%) | 56,255 (25.26%) | 116,401 (10.94%) | 106,938 (10.84%) | 9,463 (12.16%) | 636 (33.28%) | 362 (26.87%) | 274 (48.58%) | 715 (18.36%) | 661 (17.65%) | 54 (36.49%) |
| Use of antidiarrheal agents | 12,192 (1.90%) | 9,222 (2.20%) | 2,970 (1.33%) | 28,312 (2.66%) | 26,370 (2.67%) | 1,942 (2.50%) | 67 (3.51%) | 46 (3.41%) | 21 (3.72%) | 120 (3.08%) | 117 (3.12%) | 3 (2.03%) |
| Use of antibiotics | 70,112 (10.93%) | 44,177 (10.54%) | 25,935 (11.65%) | 98,570 (9.27%) | 90,212 (9.15%) | 8,358 (10.74%) | 205 (10.73%) | 156 (11.58%) | 49 (8.69%) | 365 (9.37%) | 351 (9.37%) | 14 (9.46%) |

Abbreviations: AGE, acute gastroenteritis; ICU, intensive care unit; IQR, interquartile range; SD, standard deviation

1 Index date is the first day of an episode of all-cause AGE or norovirus AGE, where all eligibility requirements are met. Unless otherwise specified, all percentages are calculated using the total number of eligible episodes as the denominator.

2 Length of stay and ICU admission are described among episodes with an acute hospitalization.

**Supplementary Table 3.** Adjusted risk ratios for acute hospitalization within three days on or after episodes for all-cause acute gastroenteritis and cause-specified norovirus acute gastroenteritis, stratified by age

| **Exposure** | **All-cause AGE^1^** | | | | | | **Norovirus AGE^2^** | | | | | |
| --- | --- | --- | --- | --- | --- | --- | --- | --- | --- | --- | --- | --- |
|  | **18-59 years** | **60-64 years** | **65-69 years** | **70-74 years** | **75-79 years** | **≥80 years** | **18-59 years** | **60-64 years** | **65-69 years** | **70-74 years** | **75-79 years** | **≥80 years** |
| **Model 1: Individual underlying medical conditions (Referent: not having a specific underlying condition)^3^** | | | | | | | | | | | | |
| Blood disorders | 1.57 (1.51, 1.64) | 1.42 (1.35, 1.50) | 1.46 (1.41, 1.52) | 1.44 (1.39, 1.49) | 1.42 (1.37, 1.47) | 1.29 (1.25, 1.33) | 1.11 (0.89, 1.38) | 1.25 (0.94, 1.65) | 1.12 (0.91, 1.37) | 1.00 (0.77, 1.30) | 1.22 (1.00, 1.48) | 1.23 (1.07, 1.40) |
| Cardiovascular disease | 2.04 (1.98, 2.11) | 1.57 (1.49, 1.65) | 1.47 (1.42, 1.53) | 1.37 (1.33, 1.42) | 1.36 (1.31, 1.41) | 1.24 (1.20, 1.28) | 1.45 (1.16, 1.82) | 1.11 (0.78, 1.58) | 1.45 (1.08, 1.95) | 1.10 (0.86, 1.41) | 1.06 (0.78, 1.45) | 1.17 (0.93, 1.48) |
| Chronic respiratory disease | 1.39 (1.35, 1.43) | 1.56 (1.50, 1.62) | 1.58 (1.53, 1.62) | 1.58 (1.54, 1.62) | 1.53 (1.49, 1.56) | 1.43 (1.40, 1.45) | 1.27 (1.06, 1.53) | 1.20 (0.92, 1.56) | 1.11 (0.94, 1.31) | 1.22 (1.03, 1.44) | 1.29 (1.12, 1.49) | 1.12 (1.01, 1.23) |
| Chronic liver disease | 1.44 (1.39, 1.49) | 1.31 (1.25, 1.37) | 1.31 (1.27, 1.36) | 1.29 (1.25, 1.33) | 1.28 (1.24, 1.32) | 1.22 (1.19, 1.26) | 1.33 (1.06, 1.67) | 1.29 (1.00, 1.67) | 1.19 (0.99, 1.43) | 1.18 (0.97, 1.44) | 1.08 (0.90, 1.30) | 1.15 (0.99, 1.33) |
| Chronic kidney disease | 1.83 (1.77, 1.90) | 1.64 (1.58, 1.71) | 1.57 (1.53, 1.62) | 1.49 (1.45, 1.53) | 1.45 (1.42, 1.49) | 1.29 (1.27, 1.31) | 1.18 (0.96, 1.45) | 1.31 (1.02, 1.67) | 1.37 (1.16, 1.61) | 1.10 (0.92, 1.32) | 1.14 (0.98, 1.32) | 1.13 (1.03, 1.24) |
| Diabetes (types I and II) | 1.28 (1.24, 1.32) | 1.11 (1.07, 1.16) | 1.08 (1.05, 1.11) | 1.14 (1.11, 1.16) | 1.12 (1.10, 1.15) | 1.09 (1.07, 1.11) | 1.00 (0.82, 1.22) | 1.08 (0.84, 1.39) | 1.18 (1.00, 1.40) | 1.07 (0.90, 1.26) | 1.14 (0.98, 1.32) | 1.14 (1.04, 1.26) |
| Obesity | 0.96 (0.93, 0.98) | 1.02 (0.98, 1.06) | 1.08 (1.05, 1.11) | 1.13 (1.10, 1.16) | 1.15 (1.12, 1.18) | 1.10 (1.07, 1.12) | 1.12 (0.94, 1.35) | 0.89 (0.69, 1.15) | 0.78 (0.64, 0.94) | 1.10 (0.94, 1.30) | 1.02 (0.87, 1.19) | 1.00 (0.89, 1.13) |
| Neurological disorders | 1.61 (1.56, 1.66) | 1.50 (1.45, 1.56) | 1.48 (1.44, 1.52) | 1.50 (1.47, 1.54) | 1.41 (1.38, 1.44) | 1.27 (1.25, 1.29) | 1.17 (0.95, 1.43) | 1.29 (1.02, 1.62) | 1.10 (0.93, 1.29) | 1.11 (0.94, 1.32) | 0.99 (0.86, 1.14) | 1.03 (0.94, 1.14) |
| Chronic gastrointestinal disease | 1.35 (1.31, 1.39) | 1.09 (1.05, 1.13) | 1.06 (1.04, 1.09) | 1.03 (1.01, 1.06) | 0.99 (0.97, 1.01) | 0.97 (0.95, 0.99) | 1.30 (1.07, 1.58) | 0.97 (0.76, 1.24) | 1.07 (0.91, 1.26) | 0.84 (0.71, 0.98) | 0.95 (0.82, 1.10) | 0.98 (0.89, 1.08) |
| Chronic immunocompromising conditions | 1.43 (1.39, 1.47) | 1.12 (1.08, 1.16) | 1.18 (1.15, 1.21) | 1.14 (1.11, 1.17) | 1.08 (1.06, 1.11) | 0.98 (0.96, 1.00) | 1.32 (1.09, 1.61) | 1.02 (0.81, 1.28) | 1.30 (1.10, 1.53) | 1.13 (0.96, 1.34) | 1.00 (0.86, 1.16) | 1.11 (1.01, 1.23) |
| **Model 2: Any underlying medical condition (Referent: no study-defined underlying conditions)^4^** | | | | | | | | | | | | |
| Any underlying medical condition | 3.91 (3.78, 4.05) | 2.67 (2.48, 2.88) | 2.19 (2.08, 2.32) | 2.01 (1.91, 2.12) | 1.70 (1.61, 1.80) | 1.36 (1.30, 1.42) | 2.62 (2.07, 3.31) | 1.85 (0.97, 3.54) | 1.79 (1.09, 2.94) | 1.16 (0.83, 1.63) | 1.46 (0.87, 2.44) | 1.25 (0.80, 1.94) |
| **Model 3: Number of underlying medical conditions (Referent: no study-defined underlying conditions)^5^** | | | | | | | | | | | | |
| Categorical number of underlying conditions |  |  |  |  |  |  |  |  |  |  |  |  |
| 1 condition | 1.43 (1.36, 1.50) | 1.10 (0.99, 1.21) | 0.98 (0.92, 1.05) | 0.95 (0.89, 1.01) | 0.86 (0.80, 0.92) | 0.90 (0.85, 0.94) | 1.11 (0.77, 1.59) | 1.30 (0.62, 2.73) | 0.99 (0.55, 1.77) | 0.85 (0.55, 1.32) | 0.99 (0.53, 1.83) | 0.99 (0.61, 1.60) |
| ≥2 conditions | 3.01 (2.89, 3.13) | 1.75 (1.61, 1.89) | 1.49 (1.40, 1.57) | 1.37 (1.30, 1.45) | 1.22 (1.15, 1.29) | 1.09 (1.04, 1.14) | 2.16 (1.61, 2.90) | 1.58 (0.79, 3.12) | 1.35 (0.80, 2.26) | 0.97 (0.68, 1.40) | 1.30 (0.77, 2.20) | 1.11 (0.71, 1.73) |
| **Model 4: Charlson-Quan comorbidity index (Referent: CCI score of 0)^6^** | | | | | | | | | | | | |
| Categorical Charlson-Quan comorbidity index |  |  |  |  |  |  |  |  |  |  |  |  |
| Score of 1 | 1.97 (1.89, 2.05) | 1.74 (1.63, 1.85) | 1.53 (1.46, 1.60) | 1.43 (1.37, 1.49) | 1.33 (1.27, 1.39) | 1.19 (1.15, 1.23) | 1.90 (1.41, 2.56) | 1.52 (0.95, 2.42) | 1.37 (0.96, 1.96) | 1.18 (0.87, 1.61) | 1.11 (0.80, 1.54) | 1.16 (0.92, 1.44) |
| Score of ≥2 | 3.79 (3.66, 3.92) | 2.44 (2.31, 2.58) | 2.09 (2.01, 2.17) | 1.96 (1.89, 2.03) | 1.79 (1.73, 1.86) | 1.52 (1.48, 1.56) | 2.88 (2.26, 3.66) | 1.52 (1.03, 2.23) | 1.64 (1.23, 2.19) | 1.30 (1.01, 1.66) | 1.30 (1.00, 1.69) | 1.21 (1.00, 1.46) |

Abbreviations: AGE, acute gastroenteritis; CCI, Charlson-Quan comorbidity index; CI, confidence interval; GEE, generalized estimating equation

1 A small proportion of all-cause AGE episodes (0.22%, n = 3,801) were excluded for having unknown or missing sex information, or other, unknown, or missing region information, resulting in 1,701,713 AGE episodes available for analysis in the GEE models.

2 A small proportion of norovirus AGE episodes (0.16%, n = 9) were excluded, resulting in 5,796 episodes for analysis.

3 Model 1: log(E[Acute hospitalization]) = β0 + β1*Blood disorders + β2*Cardiovascular disease + β3*Obesity + β4*Diabetes + β5*Chronic kidney disease + β6*Neurologic disorder + β7*Immunocompromised + β8*Chronic respiratory disease + β9*Chronic liver disease+ β10*Chronic gastrointestinal disease + β11*Age + β12*Sex + β13*Region

4 Model 2: log(E[Acute hospitalization]) = β0 + β1*Any underlying medical condition + β2*Age + β3*Sex + β4*Region

5 Model 3: log(E[Acute hospitalization]) = β0 + β1*Number of underlying conditions + β2*Age + β3*Sex + β4*Region

6 Model 4: log(E[Acute hospitalization]) = β0 + β1*Charlson-Quan comorbidity index + β2*Age + β3*Sex + β4*Region
